# Effect of Group‐Based Outpatient Physical Therapy on Function After Total Knee Replacement: Results From a Multicenter Randomized Controlled Trial

**DOI:** 10.1002/acr.23909

**Published:** 2020-05-18

**Authors:** Erik Lenguerrand, Neil Artz, Elsa Marques, Emily Sanderson, Kristina Lewis, James Murray, Tarique Parwez, Wendy Bertram, Andrew D. Beswick, Amanda Burston, Rachael Gooberman‐Hill, Ashley W. Blom, Vikki Wylde

**Affiliations:** ^1^ University of Bristol Bristol UK; ^2^ University of West of England Bristol UK; ^3^ Southmead Hospital, North Bristol NHS Trust Bristol UK; ^4^ University of Bristol and Southmead Hospital, North Bristol NHS Trust Bristol UK; ^5^ Luton and Dunstable Hospital, Luton and Dunstable University Hospital NHS Foundation Trust Luton UK; ^6^ University of Bristol and NIHR Bristol Biomedical Research Centre, University Hospitals Bristol NHS Foundation Trust Bristol UK; ^7^ University of Bristol, Southmead Hospital, North Bristol NHS Trust, and NIHR Bristol Biomedical Research Centre, University Hospitals Bristol NHS Foundation Trust Bristol UK

## Abstract

**Objective:**

To evaluate the long‐term clinical effectiveness of a novel group‐based outpatient physical therapy (PT) following total knee replacement (TKR).

**Methods:**

In this 2‐center, unblinded, superiority, randomized controlled trial, 180 patients on a waiting list for primary TKR due to osteoarthritis were randomized to a 6 session group‐based outpatient PT intervention and usual care (n = 89) or usual care alone (n = 91). The primary outcome was patient‐reported functional ability measured by the Lower Extremity Functional Scale at 12 months postoperative. Secondary outcomes included knee symptoms, depression, anxiety, and satisfaction. Questionnaires were completed preoperatively and at 3, 6, and 12 months postoperatively.

**Results:**

The mean difference in function between groups was 4.47 (95% confidence interval [95% CI] 0.20, 8.75; *P* = 0.04) at 12 months postoperative, favoring the intervention. The mean difference in function between groups decreased over time, from 8.1 points at 3 months (95% CI 3.8, 12.4; *P* < 0.001) to 5.4 (95% CI 1.1, 9.8; *P* = 0.015) at 6 months postoperative. There were no clinically relevant differences in any secondary outcomes between groups, although patients in the intervention group were more likely to be satisfied with their PT. No serious adverse events related to the intervention were reported.

**Conclusion:**

Supplementing usual care with this group‐based outpatient PT intervention led to improvements in function at 12 months after TKR, although the magnitude of the difference was below the minimum clinically important difference of 9 points. However, patient satisfaction was higher in the intervention group, and there was some evidence of clinically relevant improvements in function at 3 months.

## Introduction

Total knee replacement (TKR) is a common operation, with ~100,000 TKRs performed annually in the NHS 1, 2. The surgery is performed to reduce pain and improve function for individuals with osteoarthritis; however, 20–30% of patients with TKR report long‐term disability 3, and 20% report chronic pain 4. These poor outcomes can have a considerable negative impact on quality of life 5, 6.

**Significance & Innovations**

- This trial found that supplementing usual care with a novel group‐based outpatient physical therapy (PT) intervention led to an improvement in patient‐reported function. While there was some evidence that the short‐term improvements were clinically important, the magnitude of the benefit was not sustained in the longer term after total knee replacement.
- Patients randomized to the group‐based outpatient PT intervention were more satisfied with their PT treatment than patients in the usual care group, and the group format was considered beneficial because it provided peer support, motivation, and increased confidence.

Physical therapy (PT) is often provided to patients undergoing TKR and aims to optimize physical function. PT can be provided before surgery, in the postoperative ward, or on an outpatient basis after surgery. There is conflicting evidence of the effectiveness of preoperative PT for improving postoperative functional outcome 7, 8, 9. Postoperative inpatient PT is focused on early functional recovery and independent mobilization to ensure safe hospital discharge rather than long‐term functional improvement. Outpatient PT has been shown to improve function up to 3 months after TKR, although there is insufficient evidence to determine clinical effectiveness beyond 3 months after surgery 10.

In the UK, provision of PT after TKR is variable 11, and no definitive guidelines currently exist. Evidence is needed to guide the provision of effective PT services for patients with TKR. The primary aim of this randomized controlled trial (RCT) was to determine the clinical effectiveness of a novel group‐based outpatient PT intervention for improving long‐term function after primary TKR.

## Materials and Methods

#### Trial design

The Activity‐Orientated Rehabilitation Following Knee Arthroplasty (ARENA) study was a multicenter, pragmatic, unblinded, superiority RCT. Follow‐up assessments were performed at 3, 6, and 12 months postoperative, with a primary outcome of patient‐reported function at 12 months postoperatively. The trial was informed by a systematic review 10, survey of current practice 11, and feasibility study 12, and the protocol has been published previously 13. Reporting follows Consolidated Standards of Reporting Trials (CONSORT) guidelines (see Supplementary Material 1, available on the *Arthritis Care & Research* web site at http://onlinelibrary.wiley.com/doi/10.1002/acr.23909/abstract) and the Template for Intervention Description and Replication guidance for intervention reporting 14 (see Supplementary Material 2, available at http://onlinelibrary.wiley.com/doi/10.1002/acr.23909/abstract). A full trial‐based cost‐effectiveness analysis will be reported separately. The trial was approved by National Research Ethics Committee Southwest–Central Bristol (reference 14/SW/1144).

#### Patient and public involvement (PPI)

The trial was developed and managed in collaboration with a PPI group 15 comprising 9 patients with musculoskeletal conditions. Further details of how PPI informed the trial are reported in Supplementary Material 3, available at http://onlinelibrary.wiley.com/doi/10.1002/acr.23909/abstract, following guidance from the Guidance for Reporting Involvement of Patients and the Public 2 short form 16.

#### Participants

NHS patients were recruited from preoperative assessment clinics at 2 orthopedic centers in Bristol, UK: Southmead Hospital and Emersons Green Independent Treatment Centre. All patients provided informed written consent prior to participation. Inclusion criteria were adults on the waiting list for primary TKR due to osteoarthritis. Exclusion criteria were as follows: the inability to participate in exercise for medical reasons; being unable/unwilling to attend PT classes postoperatively; being unable/unwilling to provide informed consent; the inability to understand English; and postoperative complications within the first 2 weeks of surgery that precluded participation in PT.

#### Randomization

Participants were randomized with 1:1 treatment allocation to the intervention group or usual care group 2 weeks after TKR. Randomization with allocation concealment was conducted by the trial manager or trial administrator (KL and WB) through the Bristol Clinical Trials and Evaluation Unit using a computer‐generated code that was administered centrally and communicated via the internet. Randomization was stratified by preoperative function measured by the Lower Extremity Functional Scale (LEFS) 17 (categorized as high or low function based on mean scores from a previous study 18) and recruitment center. Due to the nature of the intervention, it was not possible to blind participants and trial personnel.

#### Usual care

At hospital discharge following TKR, patients at both centers were assessed on an individual basis by the inpatient PT team. All patients received advice on knee‐specific and functional exercises. Referral for outpatient PT was on a need‐only basis, with patients with poor range of motion or muscular weakness being further referred for outpatient PT. Criteria for referral differed between the centers and are described in Supplementary Material 4, available on the *Arthritis Care & Research* web site at http://onlinelibrary.wiley.com/doi/10.1002/acr.23909/abstract. General practitioners could also refer patients for outpatient PT as appropriate.

#### Intervention

Participants who were allocated to the intervention group received the intervention in addition to usual care. The intervention was a novel 1‐hour group‐based PT class, starting at 6 weeks after surgery and delivered on a weekly basis over 6 consecutive weeks (see Supplementary Material 2). The classes were in an NHS outpatient gymnasium and included individualized exercises within a group‐based task‐orientated exercise circuit. Classes were run on a rolling system with a maximum of 12 patients and supervised by 2 physical therapists or a physical therapist (NA) and PT technician. Delivery was in a group‐based setting, which is common in the NHS 11 and has been shown to be a cost‐effective way to deliver rehabilitation without compromising effectiveness 19.

Classes began with a short warm up, after which patients followed an exercise circuit consisting of 12 exercise stations. Ten stations were designed to increase leg strength, balance, function, and confidence using task‐related activities. Two stations were dedicated to individualized exercises, which were developed in the first class to help participants achieve their functional goals. Individualized exercises aimed to improve patients’ ability to participate in valued activities 20, to empower patients to take an active role in rehabilitation, and to increase self‐efficacy 21, 22.

Graded exercises were provided at each station to enable the patients to exercise at an intensity level suitable to their ability. Exercises progressed on an individual basis through discussion with the physical therapists (see Supplementary Material 5, available on the *Arthritis Care & Research* web site at http://onlinelibrary.wiley.com/doi/10.1002/acr.23909/abstract). Participants were given an exercise booklet in which they recorded details about their weekly progress in the class. At the end of the intervention, participants were provided with an individualized home exercise plan. Attendance at sessions was recorded, and adherence to the intervention was predefined as attendance at ≥4 sessions.

#### Outcomes

Postal questionnaires were administered preoperatively and at 3, 6, and 12 months after TKR. Participants completed additional preoperative questions on demographics, socioeconomic status, and medical comorbidities 23.

##### Primary outcome

The primary outcome was functional ability measured by the LEFS 17 at 12 months postoperative. Twelve months was the primary end point because functional outcomes after TKR start to plateau around this time 24. The LEFS is a validated 20‐item questionnaire assessing lower extremity function and difficulty in performing everyday tasks, with scores ranging from 0–80 (worst to best).

##### Secondary outcomes

The LEFS was collected at 3 and 6 months to assess lower extremity function. Knee pain, symptoms, function in daily living, function in sports and recreation, and knee‐related quality of life were assessed using the Knee Injury and Osteoarthritis Outcome Score (KOOS) 25, with each subscale score ranging from 0 to 100 (worst to best). Depression and anxiety were assessed using the Hospital Anxiety and Depression Scale (HADS) 26, with subscale scores ranging from 0 to 21 (best to worst). Patient satisfaction was assessed using the Patient Satisfaction Scale 27, with scores ranging from 25 to 100 (worst to best). Satisfaction with PT was assessed using a 5‐point Likert‐type scale (ranging from very satisfied to very dissatisfied). Self‐reported use of PT services was also captured. Health care resource use data, including data from the EuroQol 5‐domain questionnaire 28, were collected for the cost‐effectiveness analysis and will be reported separately.

##### Safety data

Participants self‐reported adverse events, and these were verified through medical records review. Serious adverse events (SAEs) were defined as any untoward medical occurrence that resulted in death, was life‐threatening, required inpatient hospitalization/prolongation of existing hospitalization, or resulted in persistent or significant disability/incapacity.

#### Process evaluation

##### Intervention

Participants who attended the classes were telephoned by a researcher 1 month after the classes and asked about their experiences of the intervention. Questions focused on satisfaction with the classes, which aspects were helpful or unhelpful, adherence to the home exercise plan, and any barriers to performing the exercises. Responses were recorded on a standardized proforma, and free‐text data were analyzed using a descriptive content analysis.

##### Trial participation

After completion of the final questionnaire, all participants were telephoned and asked about their reasons for and experiences of participation and any perceived benefits or negative aspects to participation.

#### Sample size

The minimum clinically important difference (MCID) for the LEFS is 9 scale points 17. In our feasibility study 12, the pooled SD on the LEFS score at 6 months postoperative was 18.4 points. For the purposes of the sample size calculation, a similar SD for the LEFS at 12 months after TKR was assumed. To account for the uncertainty induced by estimating parameters from a small feasibility study, the assessed sample was adjusted by an inflation factor of 1.122, a value derived from the 80% upper confidence limit of the SD estimate 29. A sample of 166 (83 participants per arm) would allow the detection of an MCID in the LEFS between trial arms at 12 months postoperatively, assuming a power of 80%, a 2‐sided 5% significance level, and accounting for an inflation factor of 1.122. In our feasibility study, the rate of missing LEFS scores at 6 months postoperative was 9% in the intervention group and 35% in the usual care group. Assuming a 35% loss to follow‐up, 256 patients would need to be recruited to include data from 166 participants in the primary analysis.

#### Statistical analysis

Analyses were performed according to the statistical analysis plan 30. Baseline characteristics were reported by trial arm using percentages, means and SDs, or medians and interquartile ranges (IQRs), as appropriate. The repeated measures of primary and secondary outcomes were plotted by trial arm.

The analyses were conducted on an intent‐to‐treat basis. The main analysis consisted of a linear mixed regression (with random intercept for patient to control for the repeated follow‐up measures) with an interaction between the intervention effect and the assessment time, adjusted for stratification variables, preoperative function, and center (with fixed effects). The use of these interaction terms allowed the estimation of time‐specific effect of the intervention on the LEFS at 3, 6, and 12 months postoperative (primary outcome). Four separate sensitivity analyses were conducted. First, the effect of clustering at surgeon level was investigated by adding an additional level to the previous linear mixed regression. Then, the analysis was adjusted for imbalanced individual characteristics between arms at baseline, followed by adjustment for whether additional PT was received. Finally, the primary outcome treatment effect was estimated using a per‐protocol approach. Given the differences in sex in TKR outcomes 31, exploratory analysis was undertaken to investigate and compare the intervention effect by sex using interaction terms. An additional analysis was conducted to investigate the effect of class size on 12 month LEFS scores using linear regression.

The analyses were conducted with and without imputation of missing primary outcome data. Missing data were imputed using multiple imputation by chained equations under a missing at random assumption stratified by randomization 32. In a sensitivity analysis of the imputation method, missing data were also imputed using the value 10% greater than the mean and 10% smaller than the mean value of the observations for each outcome. The same modeling strategy was used to investigate the intervention effect on secondary outcomes. A similar strategy was used to impute the secondary outcomes.

## Results

#### Participants

**Figure 1:**
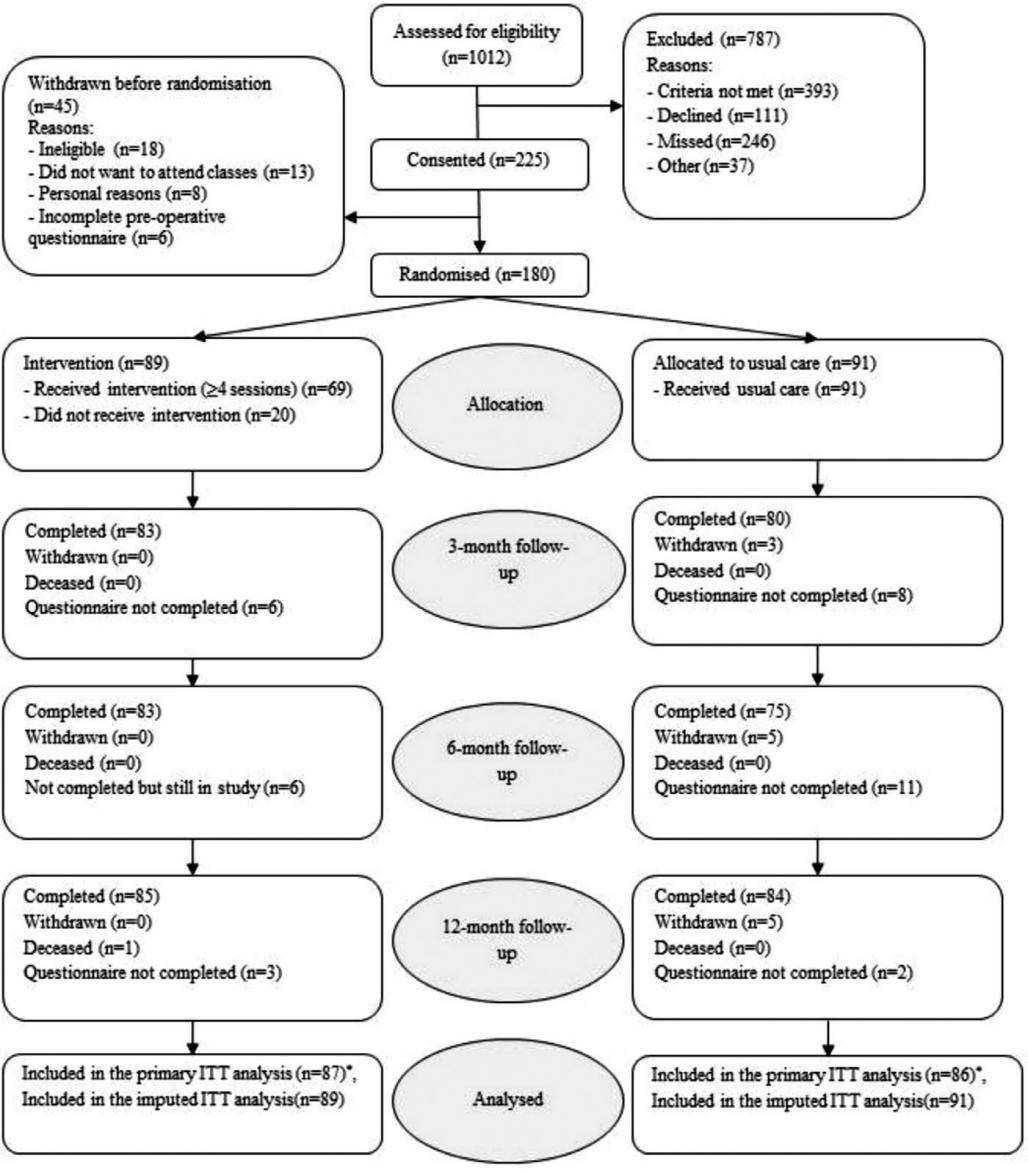
Consolidated Standards of Reporting Trials (CONSORT) flow diagram. * = number of patients who completed at least 1 postoperative Lower Extremity Functional Scale score and therefore were included in the primary mixed regression analysis; ITT = intent‐to‐treat.

Between March 2015 and March 2017, 225 patients were recruited. Of these, 45 patients withdrew prior to randomization, and 180 were randomized: 89 to the intervention and 91 to usual care (Figure 1). Questionnaires were completed by 163 participants (91%) at 3 months, 158 (88%) at 6 months, and 169 (94%) at 12 months postoperatively. The primary analysis included 173 participants who completed at least 1 postoperative LEFS questionnaire. Participants’ baseline characteristics are displayed in Table 1. Some differences in anxiety levels, education level, and working status between groups were observed and adjusted for in sensitivity analyses. Demographics of participants were similar to the national profile of patients undergoing TKR 1.

**Table 1:** Participant baseline characteristics*

	Intervention (n = 89)	Usual care (n = 91)	Overall (n = 180)
Recruitment center (Southmead)	62 (70)	64 (70)	126 (70)
Age, mean ± SD years	69 ± 9	69 ± 9	69 ± 9
Female	50 (56)	49 (54)	99 (55)
Married	55 (63)	60 (67)	115 (65)
Living with someone	60 (68)	65 (73)	125 (71)
White	84 (95)	88 (99)	172 (97)
Education beyond high school	31 (35)	22 (25)	53 (30)
Employed	25 (28)	17 (19)	42 (24)
Deprivation quintile 5 (IMD)	24 (28)	29 (33)	53 (30)
Comorbidities, median (IQR)†	2 (0.5–3)	1 (0–2)	1 (0–3)
Medial parapatellar approach‡	81 (91)	81 (89)	162 (90)
Cruciate‐retaining surgery§	53 (60)	50 (56)	103 (58)
LEFS score, mean ± SD	25 ± 15	29 ± 15	27 ± 15
KOOS total score, mean ± SD	29 ± 15	33 ± 16	31 ± 16
KOOS pain score, mean ± SD	37 ± 21	41 ± 19	39 ± 20
KOOS symptoms score, mean ± SD	39 ± 19	41 ± 20	40 ± 19
KOOS ADL score, mean ± SD	41 ± 20	44 ± 19	42 ± 20
KOOS sport/rec score, median (IQR)	5 (0–20)	10 (0–20)	5 (0–20)
KOOS QoL score, median (IQR)	25 (13–38)	25 (13–38)	25 (13–38)
HADS anxiety score, median (IQR)	7 (4–11)	5 (3–9)	6 (3–10)
HADS depression score, median (IQR)	7 (3–10)	6 (4–9)	6 (4–9)
EQ‐5D‐5L score, median (IQR)	0.6 (0.3–0.8)	0.7 (0.4–0.8)	0.6 (0.3–0.8)

* Values are the number (%) unless indicated otherwise. IMD = index of multiple deprivation; IQR = interquartile range; LEFS = Lower Extremity Functional Scale; KOOS = Knee Injury and Osteoarthritis Outcome Score; ADL = function in daily living subscale of the KOOS; sport/rec = sport and recreation subscale of the KOOS; QoL = quality of life subscale of the KOOS; HADS = Hospital Anxiety and Depression Scale; EQ‐5D‐5L = EuroQol 5‐domain, 5‐level questionnaire.

† Excluding arthritis (rheumatoid or osteoarthritis) in the number of comorbidities.

‡ All other surgical approaches were medial subvastus.

§ All other knee replacements were posterior cruciate‐sacrificing designs.

#### Intervention group

Of the 89 participants randomized to the intervention, 42 (47%) attended all 6 sessions, and 69 (78%) met the criteria for adherence. Participants attended a median of 5 classes (IQR 4–6). Reasons for nonattendance included postoperative complications, holidays, unwilling/unable to travel to the hospital, and other commitments. Classes were attended by a median of 4 participants (IQR 2–6). No effect of class size on LEFS scores at 12 months was observed (see Supplementary Material 6, available on the *Arthritis Care & Research* web site at http://onlinelibrary.wiley.com/doi/10.1002/acr.23909/abstract). During the trial period, usual care or private (self‐funded) PT (excluding the trial intervention) was received by 52% of participants in the intervention group and 58% in the usual care group (Table 2).

**Table 2:** Use of additional physical therapy (PT) services during the trial period*

Type of PT and time point	Intervention (n = 89)	Usual care (n = 91)	Overall (n = 180)
Any type of additional PT			
3	43 (48)	47 (52)	90 (50)
6	17 (19)	15 (16)	32 (18)
12	8 (9)	8 (9)	16 (9)
Any	46 (52)	53 (58)	99 (55)
1:1 hospital PT			
3	33 (37)	29 (32)	62 (34)
6	9 (10)	8 (9)	17 (9)
12	5 (6)	4 (4)	9 (5)
Any	36 (40)	35 (38)	71 (39)
PT at general practice surgery			
3	9 (10)	12 (13)	21 (12)
6	3 (3)	2 (2)	5 (3)
12	3 (3)	1 (1)	4 (2)
Any	11 (12)	12 (13)	23 (13)
Home‐based PT			
3	3 (3)	4 (4)	7 (4)
6	1 (1)	2 (2)	3 (2)
12	0 (0)	1 (1)	1 (1)
Any	3 (3)	7 (8)	10 (6)
Hydrotherapy			
3	4 (4)	3 (3)	7 (4)
6	6 (7)	3 (3)	9 (5)
12	3 (3)	3 (3)	6 (3)
Any	6 (7)	5 (5)	11 (6)
Other†			
3	1 (1)	5 (5)	6 (3)
6	3 (3)	4 (4)	7 (4)
12	1 (1)	1 (1)	2 (1)
Any	3 (3)	7 (8)	10 (6)

* Values are the number (%).

† Other includes inpatient, local community hospital, private PT, local health center, local gym, and soft tissue massage.

#### Primary analyses

**Figure 2:**
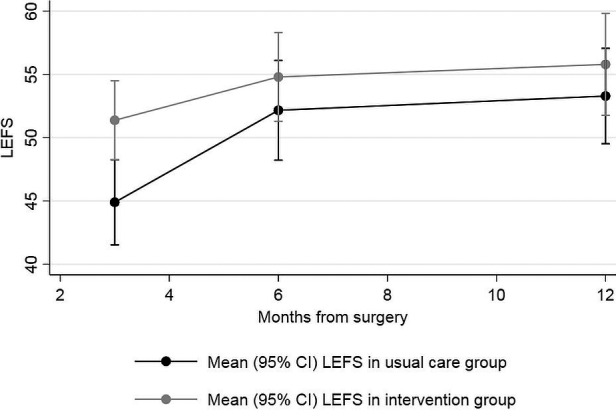
Mean Lower Extremity Functional Scale (LEFS) score with 95% confidence interval (95% CI) from randomization to 12 months postoperative for the intervention and usual care group.

A summary of all outcomes by arm and timepoint is provided in Supplementary Material 7, available at http://onlinelibrary.wiley.com/doi/10.1002/acr.23909/abstract. The mean LEFS score from randomization to 12 months postoperative by group is displayed in Figure 2. At 12 months after TKR, the mean LEFS score was 55.8 (95% confidence interval [95% CI] 51.7, 59.9) for the intervention group and 53.3 (95% CI 49.5, 57.1) for the usual care group (score distribution provided in Supplementary Material 8, available at http://onlinelibrary.wiley.com/doi/10.1002/acr.23909/abstract). The primary analyses are presented in Table 3. The primary intent‐to‐treat analysis adjusted for stratification variables suggested a difference in the mean LEFS scores at 12 months after surgery in favor of the intervention group (difference in means 4.47 [95% CI 0.20, 8.75]; *P* = 0.04). Analyses further adjusted for clustering at the surgeon level, baseline imbalances, and PT produced similar results to the primary analysis. Similar results were also found when imputing missing data (Table 3 and Supplementary Material 9, available at http://onlinelibrary.wiley.com/doi/10.1002/acr.23909/abstract). The per‐protocol analysis adjusting for stratification variables found a slighter higher difference in mean treatment effect (difference in means 6.12 [95% CI 1.60, 10.64]; *P* = 0.008). Similar results were found in the adjusted per‐protocol analysis.

**Table 3:** Intervention effect on Lower Extremity Functional Scale (LEFS) scores*

Adjustments	Intervention, no. participants	Usual care, no. participants	Difference in means	95% CI	*P*
Primary analysis (intent‐to‐treat, no imputation) of LEFS scores at 12 months					
Model 1	87	86	4.47	(0.20, 8.74)	0.040
Sensitivity analyses (intent‐to‐treat, no imputation)					
Model 2†	87	86	4.44	(0.18, 8.70)	0.041
Model 3‡			4.27	(0.10, 8.44)	0.045
Model 4			3.95	(–0.26, 8.17)	0.066
Analysis using MICE to account for missing data (intent‐to‐treat)					
Model 1	89	91	4.31	(–0.18, 8.80)	0.060
Model 2†			4.29	(–0.19, 8.77)	0.060
Model 3‡			3.93	(–0.45, 8.32)	0.079
Model 4			3.80	(–0.49, 8.10)	0.082
Per‐protocol analyses					
Model 1	69	86	6.12	(1.60, 10.64)	0.008
Model 2†			6.12	(1.60, 10.65)	0.008
Model 3‡			6.34	(1.87, 10.82)	0.005
Model 4			5.54	(1.10, 9.97)	0.014
Primary analysis (intent‐to‐treat, no imputation) of LEFS scores at 3 months					
Model 1	87	86	8.07	(3.75, 12.40)	<0.001
Primary analysis (intent‐to‐treat, no imputation) of LEFS scores at 6 months					
Model 1	87	86	5.41	(1.06, 9.77)	0.015

* Modeling strategies for analysis of LEFS scores were as follows: model 1, linear mixed regression adjusted for stratification variables, accounting for clustering within patient; model 2, linear mixed regression adjusted for stratification variables, accounting for clustering within patient and surgeon; model 3, linear mixed regression adjusted for stratification variables and baseline imbalance variables (level of education, working status, and anxiety on the Hospital Anxiety and Depression Scale [HADS] anxiety subscale), accounting for clustering within patient and surgeon; model 4, linear mixed regression adjusted for stratification variables and whether the patient had received additional physical therapy during the trial, accounting for clustering within patient and surgeon. 95% CI = 95% confidence interval; MICE = multivariate imputation via chained equations.

† The variance of the random effect associated with surgeon level was significant; this level was kept for the following sensitivity analyses.

‡ Variables that were imbalanced at baseline: level of education, working status, preoperative HADS anxiety.

Exploratory analysis of the impact of sex on LEFS scores at 12 months (see Supplementary Material 10, available at http://onlinelibrary.wiley.com/doi/10.1002/acr.23909/abstract) showed some evidence of treatment effect within males (difference in means 6.88 [95% CI 0.97, 12.79]; *P* = 0.023) but not females (2.33 [95% CI –3.19, 7.84]; *P* = 0.408). However, no evidence of a difference in the treatment effect between males and females was found (–4.55 [95% CI –12.17, 3.07]; *P* = 0.242).

#### Secondary analyses

The mean LEFS score was better in the intervention group compared to the usual care group at 3 months (difference in means 8.07 [95% CI 3.75, 12.40]; *P* < 0.001) and 6 months postoperatively (difference in means 5.41 [95% CI 1.06, 9.77]; *P* = 0.015) (Table 3). The difference in LEFS scores between groups was statistically evident at each follow‐up time point but decreased over time (difference between 3 months and 12 months in treatment effects 3.60 [95% CI 0.06, 7.15]; *P* = 0.05), with the highest treatment effect observed at 3 months postoperative. Similar results were observed in the sensitivity analyses and per‐protocol analysis (Supplementary Material 11.1 and 11.2, available on the *Arthritis Care & Research* web site at http://onlinelibrary.wiley.com/doi/10.1002/acr.23909/abstract).

There was no evidence of differences in the mean total KOOS score, KOOS subscales, HADS anxiety or depression subscales, or in Patient Satisfaction Scale scores between groups at 3, 6, or 12 (Table 4 and Supplementary Material 11.3–11.11, available at http://onlinelibrary.wiley.com/doi/10.1002/acr.23909/abstract). However, patients in the intervention group were more likely to have high satisfaction with their PT than patients in the usual care group throughout the postoperative period (Table 4 and Supplementary Material 11.12, available at http://onlinelibrary.wiley.com/doi/10.1002/acr.23909/abstract).

**Table 4:** Secondary analyses adjusted for stratification variables*

	No. patients in analysis		OR	95% CI	*P*
	Intervention	Usual care			
KOOS average score					
3 months	75	71	2.91	(0.62, 13.67)	0.177
6 months			2.52	(0.51, 12.59)	0.260
12 months			2.57	(0.52, 12.70)	0.247
KOOS QOL score					
3 months	84	77	1.05	(0.38, 2.92)	0.926
6 months			2.37	(0.81, 6.93)	0.114
12 months			1.84	(0.63, 5.37)	0.264
KOOS ADL score					
3 months	80	75	5.37	(1.37, 21.04)	0.016
6 months			3.22	(0.76, 13.59)	0.112
12 months			2.17	(0.52, 9.11)	0.291
KOOS pain score					
3 months	84	76	2.24	(0.70, 7.13)	0.173
6 months			1.64	(0.48, 5.58)	0.426
12 months			2.82	(0.79, 10.11)	0.111
KOOS sport/rec score					
3 months	82	74	2.27	(0.62, 8.32)	0.216
6 months			2.14	(0.61, 7.54)	0.235
12 months			2.30	(0.65, 8.19)	0.199
KOOS symptoms score					
3 months	85	77	1.74	(0.53, 5.71)	0.364
6 months			1.72	(0.49, 6.02)	0.393
12 months			1.89	(0.49, 6.50)	0.377
HADS anxiety score					
3 months	84	76	1.07	(0.14, 8.02)	0.946
6 months			1.61	(0.22, 11.99)	0.644
12 months			2.48	(0.36, 17.18)	0.358
HADS depression score					
3 months	84	77	0.25	(0.05, 1.39)	0.114
6 months			0.77	(0.13, 4.56)	0.769
12 months			0.57	(0.11, 2.86)	0.491
Satisfaction with surgery					
3 months	79	71	0.82	(0.30, 2.24)	0.699
6 months			1.68	(0.57, 4.98)	0.348
12 months			2.00	(0.74, 5.39)	0.170
Satisfaction with physical therapy					
3 months	84	77	0.06	(0.02, 0.20)	<0.001
6 months			0.14	(0.04, 0.47)	0.002
12 months			0.10	(0.03, 0.37)	<0.001

* Logistic regression accounting for clustering within patients for repeated measures, using contrasts and adjusted for stratification variables and preoperative outcome measures; assesses odds ratios (ORs) at 3, 6, and 12 months. 95% CI = 95% confidence interval; KOOS = Knee Injury and Osteoarthritis Outcome Score; QoL = quality of life subscale of the KOOS; ADL = function in daily living subscale of the KOOS; sport/rec = sport and recreation subscale of the KOOS; HADS = Hospital Anxiety and Depression Scale.

#### Safety

A total of 21 SAEs occurred during the trial (8 in the intervention group and 13 in the usual care group). All SAEs were deemed expected and unrelated to the intervention. Events included 14 hospital readmissions, 5 prolongations of hospital stay, 1 accident and emergency outpatient visit, and 1 death. Further details are provided in Supplementary Material 12, available at http://onlinelibrary.wiley.com/doi/10.1002/acr.23909/abstract.

#### Participant feedback

Descriptive statistics and summaries of the free‐text data are provided in Supplementary Material 13, available at http://onlinelibrary.wiley.com/doi/10.1002/acr.23909/abstract.

##### Intervention evaluation

Sixty‐eight participants completed a structured telephone survey. Participants were generally satisfied with both the task‐orientated and individualized exercises. Most thought that the class length was appropriate, although 50% of participants would have liked more classes. Participants found it helpful to have 1:1 time with a physical therapist during the classes for individualized advice and support. The group format was considered beneficial because it provided peer support, motivation, and increased confidence. While some task‐related exercises were particularly helpful to some participants, they were too easy for other patients, highlighting the difficulty in delivering an intervention catering for individuals with different levels of functional ability.

Participants found the home exercise plan useful, and most reported that they were performing their home exercises 1 month after their final class. Reasons given for discontinuation were that patients were participating in other exercises or that they felt they had good functional ability. The most common challenges in adhering to the home exercises were a lack of access to gym equipment and difficulty fitting the exercises into daily routines.

##### Trial participation

A structured telephone survey was completed by 142 participants. Altruism was the most common reason for participation, with many eager to help future patients and be involved in generating evidence to inform improvements to health care. The potential for personal benefit was also a key motivation, with participants perceiving that allocation to the classes would be beneficial. The majority of participants had a positive experience of the trial, finding it enjoyable and easy to take part in. The main suggestions for improvements included shorter questionnaires that avoided questions that were perceived to be irrelevant and repetitive.

## Discussion

This is the first trial to evaluate whether group‐based outpatient PT can improve patient‐reported function up to 12 months after TKR in an NHS setting. Supplementing usual care with a novel group‐based outpatient PT intervention resulted in better patient‐reported function at 3, 6, and 12 months after TKR. However, the difference in function at 12 months was below the MCID, suggesting the intervention may not result in a clinically important improvement in function. However, the intervention was safe, associated with higher patient satisfaction, and there was some evidence of a clinically important short‐term benefit at 3 months postoperatively.

This project was informed by a robust series of projects, including a feasibility study 12, in line with Medical Research Council guidance on complex interventions 33. PPI activities guided the design and management of the trial to ensure that the research was relevant and acceptable to patients. Patients were recruited from an NHS independent treatment center and an elective orthopedic center, thereby increasing the generalizability of the results. This was a pragmatic trial with patient eligibility criteria, intervention delivery, and nonstandardized usual care designed to reflect how the intervention would be delivered if implemented within usual NHS care. However, limitations of the trial should also be acknowledged when interpreting the results. As with many PT trials 34, blinding of the intervention was not possible, which could have led to an overestimation of the treatment effect 35. While this risk of bias may have influenced the positive short‐term effects, the decrease in treatment effect over time would have rendered this bias less meaningful.

Another potential limitation is our use of the MCID threshold of the LEFS. Using the MCID allows a more meaningful evaluation of the clinical relevance of the results rather than simply interpreting the results based on statistical significance. However, the MCID for the LEFS was derived from patients with a variety of lower extremity musculoskeletal conditions 17, which may limit the applicability to patients with TKR. It should also be acknowledged that findings from our trial only relate to the specific PT intervention that we evaluated. However, other trials have reported similar findings. Since the publication of a systematic review that found insufficient evidence to evaluate the long‐term effectiveness of postdischarge PT 10, relevant trials have been published. An Australian trial found that an outpatient exercise program did not improve patient‐reported outcomes at 1 year after TKR compared to usual care 36. Another Australian trial found that 10 days of postdischarge inpatient PT and a monitored home exercise program did not improve walking ability at 6 months after TKR compared with a home exercise program only 37.

There are a number of potential reasons why our intervention was only associated with a small improvement in function at 12 months after TKR. First, we limited the treatment to 6 classes to optimize the feasibility of the intervention being implemented into usual NHS practice if found to be effective. It is possible that a more intensive intervention may have had a beneficial effect; however, previous studies that have evaluated more intensive interventions have found similar results 36, 37, 38. Also, no follow‐up support was provided to participants for their home exercise program, which may have resulted in participants not continuing an adequate amount of home exercises and contributed to the short‐term benefits not being sustained in the longer term. Second, both groups had access to usual care and private PT because we wanted to assess the effectiveness of the intervention as implemented within the NHS setting. Very few trials compare a PT intervention to no care 10, 39, and the purpose of our trial was to evaluate if the addition of group‐based PT to usual care could improve patient outcomes. This resulted in approximately one‐half of participants in both arms using usual care or private PT during the follow‐up, although PT usage was balanced between trial arms, and adjustment in sensitivity analyses produced similar results. Third, adherence to rehabilitation interventions is a common issue 40. Similar to a previous study 36, only one‐half of participants randomized to the intervention group attended all the classes. Per‐protocol analysis of the 78% of participants who met the prespecified adherence criteria found a larger treatment effect, and the 95% CIs suggest that the true difference at 12 months could reach a clinically meaningful level. This suggests a possible dose effect, which has been found previously 41, and that attending <6 sessions may not provide patients with an adequate intervention level to change outcomes. Higher‐dose PT is unlikely to be pragmatic in the NHS; however, supplementing weekly classes with guidance on home exercises could be beneficial and warrants further research. Fourth, we used a patient‐reported outcome measure to capture patients’ experiences of their function, rather than objective measures or performance tests to evaluate their actual functional ability, because performance tests may not capture limitations experienced during important daily activities 42. However, self‐reported function is strongly influenced by pain 43, 44, and the small difference in outcomes between groups may be because the intervention was not designed to reduce pain. The intervention may have shown a larger effect on objective outcomes or performance measures, which are less influenced by pain and more sensitive to changes in function 43, 44. Further research is warranted.

In conclusion, addition of group‐based outpatient PT to usual NHS care led to improvements in function at 12 months after TKR, although the magnitude of the difference did not reach a clinically meaningful level. However, patient satisfaction was higher in the intervention group, and there were clinically relevant improvements in function at 3 months. This suggests that there is early benefit from PT with the potential for longer term benefit. Recommendations for future research include evaluating the optimal mode of PT delivery to maximize patient benefit, including intensity, duration, progression, and support with home exercises. Our findings add to the evidence on the effectiveness of group‐based outpatient PT to guide decisions by clinicians and patients and to inform commissioning of services to ensure that patients receive optimal PT after TKR.

## Author Contributions

All authors were involved in drafting the article or revising it critically for important intellectual content, and all authors approved the final version to be submitted for publication. Dr. Wylde had full access to all of the data in the study and takes responsibility for the integrity of the data and the accuracy of the data analysis.

### Study conception and design

Lenguerrand, Artz, Marques, Murray, Parwez, Beswick, Burston, Gooberman‐Hill, Blom, Wylde.

### Acquisition of data

Lewis, Bertram.

### Analysis and interpretation of data

Lenguerrand, Sanderson, Wylde.

## Supporting information

Supplementary Material
