# Supplementary material for: Effect of Group‐Based Outpatient Physical Therapy on Function After Total Knee Replacement: Results From a Multicenter Randomized Controlled Trial
Source: Arthritis Care Res (Hoboken). 2020 May 18;72(6):768–77. doi: 10.1002/acr.23909 (PMC7317425; doi:10.1002/acr.23909)
Supplement: Supplementary file 1 — Supplementary Material [file ACR-72-768-s001.doc]

ARENA Supplementary materials

Contents

[Supplementary material 1: CONSORT checklist 2](#__RefHeading___Toc2583417)

[Supplementary material 2: Summary of the ARENA trial intervention using TiDIER criteria 4](#__RefHeading___Toc2583418)

[Supplementary material 3: Patient and public involvement in the ARENA trial 8](#__RefHeading___Toc2583419)

[Supplementary material 4: Further details on usual care 10](#__RefHeading___Toc2583420)

[Supplementary material 5: Further details on progression of exercises 11](#__RefHeading___Toc2583421)

[Supplementary material 6: Class size effect on the LEFS at 12 months post-operative 13](#__RefHeading___Toc2583422)

[Supplementary material 7: Summary of all outcomes by arm and timepoint 14](#__RefHeading___Toc2583423)

[Supplementary material 8: Histogram of LEFS at 12 months by trial arm 15](#__RefHeading___Toc2583424)

[Supplementary material 9: Primary analysis^1^ of LEFS at 12 months – additional missing data analyses 16](#__RefHeading___Toc2583425)

[Supplementary material 10: Investigation of gender-specific effect of the intervention effect 17](#__RefHeading___Toc2583426)

[Supplementary material 11.1: LEFS at 3 months: Sensitivity and per protocol analyses 19](#__RefHeading___Toc2583427)

[Supplementary material 11.2: LEFS at 6 months: Sensitivity and per protocol analyses 20](#__RefHeading___Toc2583428)

[Supplementary material 11.3: KOOS Total: Sensitivity and per protocol analyses 22](#__RefHeading___Toc2583429)

[Supplementary material 11.4: KOOS QOL: Sensitivity and per protocol analyses 24](#__RefHeading___Toc2583430)

[Supplementary material 11.5: KOOS ADL: Sensitivity and per protocol analyses 25](#__RefHeading___Toc2583431)

[Supplementary material 11.6: KOOS Pain: Sensitivity and per protocol analyses 26](#__RefHeading___Toc2583432)

[Supplementary material 11.7: KOOS Sport/Rec: Sensitivity and per protocol analyses 27](#__RefHeading___Toc2583433)

[Supplementary material 11.8: KOOS Symptoms: Sensitivity and per protocol analyses 28](#__RefHeading___Toc2583434)

[Supplementary material 11.9: HADS Anxiety: Sensitivity and per protocol analyses 29](#__RefHeading___Toc2583435)

[Supplementary material 11.10: HADS Depression: Sensitivity and per protocol analyses 31](#__RefHeading___Toc2583436)

[Supplementary material 11.11: Satisfaction with surgery outcome: Sensitivity and per protocol analyses 32](#__RefHeading___Toc2583437)

[Supplementary material 11.12: single item Satisfaction with physiotherapy: Sensitivity and per protocol analyses 34](#__RefHeading___Toc2583438)

[Supplementary material 12: Serious adverse event data 36](#__RefHeading___Toc2583439)

[Supplementary material 13: Process evaluation data 37](#__RefHeading___Toc2583440)

# Supplementary material 1: CONSORT checklist

| Section/Topic | Item No | Checklist item | Reported on page No |
| --- | --- | --- | --- |
| Title and abstract | | | |
|  | 1a | Identification as a randomised trial in the title | 1 |
|  | 1b | Structured summary of trial design, methods, results, and conclusions (for specific guidance see CONSORT for abstracts) | 3-4 |
| Introduction | | | |
| Background and objectives | 2a | Scientific background and explanation of rationale | 6 |
|  | 2b | Specific objectives or hypotheses | 6 |
| Methods | | | |
| Trial design | 3a | Description of trial design (such as parallel, factorial) including allocation ratio | 6 |
|  | 3b | Important changes to methods after trial commencement (such as eligibility criteria), with reasons | N/A |
| Participants | 4a | Eligibility criteria for participants | 7 |
|  | 4b | Settings and locations where the data were collected | 7-9 |
| Interventions | 5 | The interventions for each group with sufficient details to allow replication, including how and when they were actually administered | 8-9, Supplementary material 2 & 5 |
| Outcomes | 6a | Completely defined pre-specified primary and secondary outcome measures, including how and when they were assessed | 9-10 |
|  | 6b | Any changes to trial outcomes after the trial commenced, with reasons | N/A |
| Sample size | 7a | How sample size was determined | 10-11 |
|  | 7b | When applicable, explanation of any interim analyses and stopping guidelines | N/A |
| Sequence generation | 8a | Method used to generate the random allocation sequence | 7-8 |
|  | 8b | Type of randomisation; details of any restriction (such as blocking and block size) | 7-8 |
| Allocation concealment mechanism | 9 | Mechanism used to implement the random allocation sequence (such as sequentially numbered containers), describing any steps taken to conceal the sequence until interventions were assigned | 7-8 |
| Implementation | 10 | Who generated the random allocation sequence, who enrolled participants, and who assigned participants to interventions | 7-8 |
| Blinding | 11a | If done, who was blinded after assignment to interventions (for example, participants, care providers, those assessing outcomes) and how | N/A |
|  | 11b | If relevant, description of the similarity of interventions | 8-9 |
| Statistical methods | 12a | Statistical methods used to compare groups for primary and secondary outcomes | 11 |
|  | 12b | Methods for additional analyses, such as subgroup analyses and adjusted analyses | 11 |
| Results | | | |
| Participant flow (a diagram is strongly recommended) | 13a | For each group, the numbers of participants who were randomly assigned, received intended treatment, and were analysed for the primary outcome | Figure 1 |
|  | 13b | For each group, losses and exclusions after randomisation, together with reasons | Figure 1 |
| Recruitment | 14a | Dates defining the periods of recruitment and follow-up | 12 |
|  | 14b | Why the trial ended or was stopped | N/A |
| Baseline data | 15 | A table showing baseline demographic and clinical characteristics for each group | Table 1 |
| Numbers analysed | 16 | For each group, number of participants (denominator) included in each analysis and whether the analysis was by original assigned groups | Tables 3 & 4, Supplementary materials |
| Outcomes and estimation | 17a | For each primary and secondary outcome, results for each group, and the estimated effect size and its precision (such as 95% confidence interval) | Tables 3 & 4, Supplementary materials |
|  | 17b | For binary outcomes, presentation of both absolute and relative effect sizes is recommended | N/A |
| Ancillary analyses | 18 | Results of any other analyses performed, including subgroup analyses and adjusted analyses, distinguishing pre-specified from exploratory | Supplementary materials |
| Harms | 19 | All important harms or unintended effects in each group (for specific guidance see CONSORT for harms) | 14, Supplementary material 12 |
| Discussion | | | |
| Limitations | 20 | Trial limitations, addressing sources of potential bias, imprecision, and, if relevant, multiplicity of analyses | 16 |
| Generalisability | 21 | Generalisability (external validity, applicability) of the trial findings | 15 |
| Interpretation | 22 | Interpretation consistent with results, balancing benefits and harms, and considering other relevant evidence | 15-18 |
| Other information | | |  |
| Registration | 23 | Registration number and name of trial registry | 4 |
| Protocol | 24 | Where the full trial protocol can be accessed, if available | 7 |
| Funding | 25 | Sources of funding and other support (such as supply of drugs), role of funders | 19 |

# Supplementary material 2: Summary of the ARENA trial intervention using TiDIER criteria

| **TiDIER Criteria** | **Description of ARENA trial intervention** |
| --- | --- |
| **Brief name** | |
| Name | ARENA (activity-orientated rehabilitation following knee arthroplasty): Outpatient group-based physiotherapy with an individualised component for patients after total knee replacement |
| **Why** | |
| Rationale, theory, or goal of the elements essential to the intervention | Previous research has demonstrated the benefits of task-orientated, functional exercises [1] and the importance of improving patients’ ability to participate in ‘valued activities’ [2 3]. Task-orientated exercises have been found to be more effective than traditional exercises, such as range of motion exercises, in improving function and participation after total knee replacement [1] and other conditions such as stroke [4]. The inclusion of individualised exercises aims to address patients’ expectations, empower people to take an active role in rehabilitation, and increase self-efficacy [5 6]. Delivery is in a group-based setting, which can be a cost-effective way to deliver rehabilitation without compromising effectiveness [7-9]. There is currently no consensus on the optimal treatment frequency or number of sessions for physiotherapy after total knee replacement [10]. The intensity of our intervention was informed by previous research [11], combined with the need to develop an intervention that would be deliverable within the NHS if proven to be effective. |
| **What** | |
| Materials | Training manual: Comprehensive guidance covering the set up and delivery of the intervention, role of the physiotherapist during the intervention, details of the exercises within the intervention, development of the home exercise programme, procedures to be followed for non-attendance of participants and staff, emergency and security procedures, and reporting of adverse events.  Activity goal form: Before attending the physiotherapy classes, participants were asked to complete an activity goal form which involved identifying two functional goals that they would like to achieve.  Exercise booklet: Used by participants to record details about their weekly progress in the class, including the number of sets and duration at each exercise station.  Personalised home exercise plan: Advice on continuation of exercises at home after completion of the classes.  Gym equipment: Plinths x3, pillows x6, multi-gym, gym ball x2, static exercise bike, treadmill, cross-trainer, stepper machine, air stability wobble cushion, multi-directional wobble board, uniplanar wobble cushion, large chair, large pressure relief cushion, large gym mat x2, large ball (football / basketball / medicine ball), small ball (sponge ball / tennis ball), steps / stairs, bat and ball, small plastic traffic cone, skittles, timer  All paperwork are available from the research team on request |
| Procedures | **Warm up**  Each class started with a 5-minute warm up. This involved participants mobilising around the gym circuit at a steady pace, interspersed with a selection of gentle upper limb and lower limb movements including shoulder circumduction, elbow flexion and extension, hip and knee flexion, and ankle circumduction exercises at the discretion of the physiotherapist.  **Class overview**  After the warm up, patients followed a simple exercise circuit. The exercise circuit involved 12 exercise stations, with 10 stations for task-orientated exercises and 2 stations dedicated to individualised exercises. Four minutes was allocated to each station to provide patients with sufficient time to carry out the exercises at their own pace. Participants did not have to exercise for the full four minutes and could rest or pace the activity accordingly. Thirty seconds resting time was allocated between stations. The maximum capacity for the classes was 12 patients to allow one patient to exercise per exercise station.  **Task-orientated exercise stations**  Bed-based exercises: Low grade exercises including knee flexion and extension range of motion (2 x 8 repetitions), quadriceps strengthening (2 x 8 repetitions), hamstring strengthening (2 x 8 repetitions), quadriceps stretching (2 x 5 repetitions), and hamstrings stretching (2 x 5 repetitions). Progressions include increasing number of repetitions, changing position, and addition of resistance bands or ankle weights.  Getting in/out of bed: Practice turning from back to side and to sitting, stand from sitting, return to sitting and then lying (2 x 5 repetitions). Progression includes bridging then sit to stand.  Balance: Single leg stance (3 x 30 seconds) and wobble board (3 x 30 seconds). Progressions including increasing duration and including upper limb actions such as throwing, catching and reaching.  Stairs: Stepping up and down on stairs of varying height (3 x 8 step-ups). Progression includes using higher step.  Walking: Straight line walking. Progressions including from aided to unaided, side stepping, walking over uneven surfaces, walking carrying objects.  Squatting: Mini and semi-squats (3 x 8 repetitions). Squats can be performed with the assistance of chairs and gym ball. Progressions including increasing the depth of squat and crouching.  Cycling: Static bike (1 minute cycling followed by 30 seconds rest and then repeat). Progressions include increasing resistance and duration of cycling.  Kneeling: Replicating digging action using stepper (3 x 8 repetitions) with progression including increasing resistance of stepper. Kneeling onto cushioned or hard surfaces (3 x 8 repetitions) with progression including full kneeling and high kneeling. Activities to desensitise the knee joint such as light pressure using different textures (30-60 seconds).  Lunges: Mini lunges (2 x 8 repetitions). Progressions include depth of lunge, lunge walking and lunge to bowling or picking up objects.  Treadmill/cross-trainer: Straight line walking. Progressions including increase in speed and incline of treadmill, use of cross-trainer  **Individualised exercise stations**  Before attending the physiotherapy classes, participants were asked to complete an activity goal form which involved identifying two functional goals that they would like to achieve. In their first class, each participant had a discussion with the physiotherapist about these goals to develop two individualised exercises which they began in their second class.  **Grading and progression**  The physiotherapy staff advised on progression/regression or adaptation of exercises as appropriate to each individual participant. A selection of graded exercises was provided at each station to enable the patients to exercise at a level suitable to their ability. During weeks 2-6, the exercises were progressed on an individual basis through discussion with the physiotherapists.  **Home exercise programme**  Towards the end of the intervention, the physiotherapist spent time with each participant to develop their home exercise programme. Every participant was provided with an individualised written plan detailing the key exercises to continue at home. Advice on number of repetitions and how often the exercises should be performed was provided, tailored to the participant’s individual ability. |
| **Who provided** | |
| For each category of intervention provider, describe their expertise, background and any specific training given | Each class was staffed by two members of staff, one of which was a HCPC registered chartered physiotherapist with experience in musculoskeletal physiotherapy. The chartered physiotherapist was the lead for the class and responsible for overseeing the patients care during the exercise class. The lead physiotherapist was also responsible for reporting any adverse events to the research team or discussing medical queries with the patient. The second member of staff was a physiotherapy assistant or technician, and was responsible for assisting in the set-up and delivery of the exercise class including discussing progression with the participant. For the classes when a physiotherapy assistant or technician was not available, the second member of staff was a chartered physiotherapist. |
| **How** | |
| Describe the modes of delivery of the intervention | Face-to-face classes were delivered in a group setting, with a maximum of 12 patients. Classes were run on a rolling system so that new patients could join the classes each week. New participants were asked to arrive at the exercise class 15 minutes before the start of the class so that the lead physiotherapist could introduce the gymnasium and exercise stations, and discuss individual medical conditions, the exercise booklet and collect the activity goal form. |
| **Where** | |
| Describe the type of location where the intervention occurred | Large outpatient hospital gymnasium with necessary gym equipment. |
| **When and how much** | |
| Describe the number of times the intervention was delivered and over what period of time | Participants began the classes at six weeks after their total knee replacement. Classes were weekly for six weeks. Each class was one hour long. Participants were invited to attend a total of six hours of classes. |
| **Tailoring** | |
| If the intervention was planned to be personalised, titrated or adapted, then describe what, why, when, and how. | Individualised exercise stations: Before attending the physiotherapy classes, participants were asked to complete an activity goal form which involved identifying two functional goals that they would like to achieve. In their first class, each participant had a discussion with the physiotherapist about these goals to develop two individualised exercises which they began in their second class.  Grading and progression: The physiotherapy staff advised on progression/regression or adaptation of exercises as appropriate to each individual participant. A selection of graded exercises was provided at each station to enable the patients to exercise at a level suitable to their ability. During weeks 2-6, the exercises were progressed on an individual basis through discussion with the physiotherapists.  Home exercise programme: Towards the end of the intervention, the physiotherapist spent time with each participant to develop their home exercise programme. Every participant was provided with an individualised written plan detailing the key exercises to continue at home. Advice on number of repetitions and how often the exercises should be performed was provided, tailored to the participant’s individual ability. |
| **Modifications** | |
| If the intervention was modified during the course of the study, describe the changes | Not applicable. |
| **How well** | |
| If intervention adherence or fidelity was assessed, describe the extent to which the intervention was delivered as planned. | The physiotherapist recorded participant attendance at the exercises class on a register. Non-attendees were telephoned and asked if they would be willing to provide their reasons for non-attendance. Reasons were recorded on a standardised proforma. Adherence to the intervention was predefined as attendance at ≥4 sessions.  Adherence to the home exercise programme was assessed at one month after the final class during a structured telephone discussion with a member of the research team.  Intervention adherence is reported in the article. |

**References**

1. Minns Lowe CJ, Barker KL, Dewey M, et al. Effectiveness of physiotherapy exercise after knee arthroplasty for osteoarthritis: systematic review and meta-analysis of randomised controlled trials. BMJ 2007;**335**(7624):812.

2. Wylde V, Cavendish V, Learmonth I, et al. Values for function in osteoarthritis and rheumatoid arthritis patients. Rheumatology (Oxford) 2006;**45**(Supplement 1):71.

3. Wylde V, Livesey C, Blom AW. Restriction in participation in leisure activities after joint replacement: an exploratory study. Age Ageing 2012;**41**(2):246-9.

4. Van Peppen RP, Kwakkel G, Wood-Dauphinee S, et al. The impact of physical therapy on functional outcomes after stroke: what's the evidence? Clin Rehabil 2004;**18**(8):833-62.

5. Orbell S, Johnston M, Rowley D, et al. Self-efficacy and goal importance in the prediction of physical disability in people following hospitalization: a prospective study. Br J Health Psychol 2001;**6**(Pt 1):25-40.

6. Westby MD, Backman CL. Patient and health professional views on rehabilitation practices and outcomes following total hip and knee arthroplasty for osteoarthritis:a focus group study. Bmc Health Services Research 2010;**10**:119.

7. Mitchell C, Walker J, Walters S, et al. Costs and effectiveness of pre- and post-operative home physiotherapy for total knee replacement: randomized controlled trial. J Eval Clin Pract 2005;**11**(3):283-92.

8. Hurley MV, Walsh NE, Mitchell HL, et al. Economic evaluation of a rehabilitation program integrating exercise, self-management, and active coping strategies for chronic knee pain. Arthritis Rheum 2007;**57**(7):1220-9.

9. Coulter CL, Weber JM, Scarvell JM. Group physiotherapy provides similar outcomes for participants after joint replacement surgery as 1-to-1 physiotherapy: a sequential cohort study. Arch Phys Med Rehabil 2009;**90**(10):1727-33.

10. Westby MD, Brittain A, Backman CL. Expert consensus on best practices for post-acute rehabilitation after total hip and knee arthroplasty: a Canada and United States Delphi study. Arthritis Care Res (Hoboken) 2014;**66**(3):411-23.

11. Artz N, Elvers K, Minns Lowe C, et al. Effectiveness of physiotherapy exercise following total knee replacement: systematic review and meta-analysis. BMC Musculoskeletal Disorders 2015;**16**(15).

# Supplementary material 3: Patient and public involvement in the ARENA trial

**Aim**

To optimise research design, management and dissemination and to ensure the research was acceptable to patients and remained relevant to the needs of patients.

**Methods**

This trial was conducted in collaborated with the Patient Experience Partnership in Research (PEP-R) group at the Musculoskeletal Research Unit. PEP-R is a dedicated, specialised PPI group comprising nine patients with musculoskeletal conditions, most of whom have had joint replacement. Since 2010, PEP-R has met in 2-hour forum sessions every two months and is supported by an experienced PPI co-ordinator (AB). The research team met with PEP-R five times during the project to discuss trial design, management and dissemination.

During the course of the trial, eight TSC meetings were held, with two update reports by e-mail. Two patient representatives and the PPI co-ordinator were members of the Trial Steering Committee (TSC). Patient representatives were sent paperwork before and after the meetings, including a 'jargon' buster to assist their understanding of any research or medical terminology used.

**Study results: PEP-R meetings**

The patient information leaflet and questionnaire were discussed with PEP-R in September 2014. Suggestions for improvements on the patient information leaflet included adding more detail about the home exercise plan, increasing the estimated duration of questionnaire completion from 30 minutes to 30-45 minutes, and reordering the sections so that usual care was described before the intervention. All these suggestions were actioned. For the questionnaire, PEP-R suggested simplifying the front cover and re-ordering the questions so that knee-related questions were first (as these are most relevant to participants) and socioeconomic questions were last (as people may find these questions intrusive). These suggestions were actioned.

The research team met with PEP-R again in November 2014. The first half of the meeting was a training session to discuss the processes and timelines involved in applying for a NIHR grant, using ARENA as an example. In the second half, the ARENA exercise booklet was discussed, particularly the use and depiction of a traffic light system to help patients pace themselves (red=too much exercise, amber=amount right, green=could have done more). PEP-R members thought this was a useful tool but suggested that it needed to be made clearer that the aim was to reach amber, not green, and this was subsequently added to the booklet. There was also a discussion around the use of pain medications prior to the exercises classes and PEP-R members thought that it was appropriate to recommend that people continue with their usual pain medication rather than take some before the classes. This was subsequently changed in the booklet.

In February 2016, PEP-R discussed the change of venue of the classes from a large hospital (Southmead Hospital) to a community hospital (Cossham Hospital). This proposed move was based on feedback from patients that they did not want to participate because they did not want to attend classes at Southmead Hospital due to parking issues. PEP-R thought that moving the classes to Cossham Hospital would be an improvement and more appealing to most people because parking is easier. The letter about the change of class venue that was to be sent to participants was also reviewed. PEP-R thought that it would be useful to include a map of Cossham with the letter, and this was actioned. They also suggested that the class should be held mid-morning. This was because it would allow people time to get up and have breakfast before exercising. Early morning would be difficult as the patient representatives described how their knees could be stiff early in the morning, but that knee function was usually optimal around 10am-2pm.

In March 2018, a discussion was held about how to disseminate the findings to reach a broad audience. PEP-R provided many useful suggestions including hospital magazines, local and national media, charity newsletters, and contacting commissioners. They suggested websites, posters and YouTube videos as methods of dissemination.

The final meeting was held in September 2018, and a plain English summary of the trial findings was circulated to PEP-R members before the meeting. The findings were discussed, alongside the graph of the results (Figure 2 in the manuscript). The group were surprised that the study found that outpatient physiotherapy was not beneficial for long-term mobility but agreed with the findings from the process evaluation that group-based exercise has social benefits and is enjoyable. A discussion was held about how future research could facilitate the positive aspects of the intervention outside of NHS physiotherapy, such as social prescribing of exercise and increased engagement with community-based exercise groups.

**Study results: TSC patient representatives**

An example of how the TSC patient representatives inputted is through the design of a substantial amendment to maximise follow-up questionnaire completion. The following suggestions were made and implemented:

1.) Sending a questionnaire pre-notification card to prepare patients to receive the questionnaire

2.) Including tea and coffee sachets with the study questionnaires to ensure the participants knew their contribution was valued

3.) Amending questionnaire cover letters to clarify that the research team would phone back participants who contacted the team to ensure cost was not a barrier to participants

4.) Sending thank you cards to participants when they were 9 months post-operative.

**Discussion and conclusions**

PPI had meaningful and positive impact on the ARENA trial, including on the design of study documents, delivery of the intervention, follow-up and dissemination.

**Reflective perspective**

Regular engagement with the PEP-R group ensured that the members were engaged in the project and genuinely interested in the findings. The presence of the PPI co-ordinator at the TSC meetings was particularly useful to ensure that discussions were conducted in language that enabled all members to actively participate.

# Supplementary material 4: Further details on usual care

Further details of referral for outpatient physiotherapy at each of the two centres are provided below:

**Emersons Green Independent Treatment Centre**

Any of the following criteria would trigger a referral for outpatient physiotherapy:

1.) Poor range of motion (not achieving full extension or 90^O^ flexion) or poor static quadriceps contraction after 2-3 days of inpatient physiotherapy

2.) Significant muscle weakness (no objective measure used to assess this)

3.) Patients who need extra input for medical reasons or

4.) Post-operative complications, such as foot drop

**Southmead Hospital**

There were a number of different orthopaedic teams at Southmead Hospital and criteria for referral for post-discharge physiotherapy was on a needs basis determined by the individual clinician. Although there were no clearly defined criteria for referral, the generally policy was that patients considered to have quadriceps weakness (no objective measure used to assess this), physically active jobs or considered to require greater input at home would be referred to appropriate physiotherapy services such as outpatient or community-based physiotherapy.

# Supplementary material 5: Further details on progression of exercises

Each exercise in the class had a number of progressions to enable the participants to be challenged appropriately and safely. Primarily, progressions were based on increasing the intensity of the exercise, such as increasing sets, repetitions, duration or by changing the difficulty of the task. The initiation of such progression was through successful achievement of the exercise intensity and discussion with the supervising physiotherapist. Similarly, if participants were unable to achieve exercise intensities successfully, they were regressed by reducing the number of sets, repetitions, duration or by adapting the exercise to reduce the difficulty. As the intervention was designed to provide a range of functional exercises tailored to each participant’s capability, a uniform progression with each individual exercise was not always possible and participants were progressed according to capability and choice.

| **Exercise** | **Description** | **Progressions / regressions** |
| --- | --- | --- |
| Bed-based exercises | Active range of motion exercises for knee flexion and extension in supine lying or long sitting. Initial target of 2 sets of 8 repetitions were prescribed.  Quadriceps strengthening (open chain) and  hamstring strengthening (open chain). Initial target of 2 sets of 8 repetitions with no weight.  Quadriceps and hamstring stretching. Patients advised to hold for 2 sets of 15-30 secs depending on tolerance. | Range of motion exercises were progressed by increasing repetitions, sets and range of motion accordingly. Progressed to 2 sets of 10, 12, or 15 depending upon successful completion of the exercise.  Range of motion exercises were progressed by increasing repetitions, sets and adding resistance with small ankle weights. Progressed to either 2 sets of 10, or 3 sets of 8 with additional resistance with 0.5Kg ankle weight.  Static quadriceps stretch in standing. Static hamstring stretch (90:90 position) in supine lying. |
| Getting in and out of bed | Participants practiced turning from supine to side lying and then to sitting (2 x 5 repetitions). Participants could choose the direction (left or right) to get out of bed depending on their normal habits. Initial target of 2 sets of 5 repetitions of lying to sitting. | Progressions included increasing the number sets and repetitions of each exercise as tolerated, standing up from sitting and then returning to sitting and then lying, bridging in supine lying and then from lying to sitting and then to stand. |
| Balance exercises | Single leg stance (operated side). Initial exercise was 3 x 30 seconds. | Progression included duration of stance time, repetitions, sets and difficulty. Difficulty was also progressed to performing exercise with eyes closed to a maximum of 60 seconds, or by throwing and catching a ball. Additional progression was to challenge stability further with the use of a wobble board, and then to single leg stance on wobble board. |
| Stair climbing | Participants utilised portable stair unit to practice ascending and descending stairs. Initial exercise consisted of 3 sets of 8 step ups (on both legs). | Progression of stair climbing included increasing sets, repetitions, stair height and difficulty of the task, according to the patient’s ability and confidence in completing the task. Progressions were to ascend the 5 small stairs steps and descend holding on to a hand rail, reducing support from hand rails and ascending and descending deeper stairs with and without the use of rail support. |
| Walking practice | Participants practiced walking in a straight line along a 5m area of gym space. Participants were provided with gait re-education from the supervising therapists. | Progressions in walking included reduction of walking aids as appropriate, duration of walking time, side-stepping, walking over uneven surfaces, walking around cones, figure of 8, and walking whilst carrying a bat and ball. Durations were set according to participant ability. Patients were progressed from aided to unaided gait following discussion with the physiotherapist. |
| Squats | Participants performed squats using wall rails for support. Initial target was 3 sets of 8 repetitions. | Progressions included increasing sets, repetitions, depth of squat (mini-squat to semi-squat position) and decreasing support from wall rail. |
| Cycling | Static bike (2x 1 minute with 30 sec rest) | Progressions included increasing duration and reduced rest period, increasing range of motion from semi-revolution of the pedals to full revolution, increasing the resistance level on the bike, and increasing the distance (km). |
| Gardening and kneeling | Exercise station to practice kneeling and simulated digging using a resistance stepper machine. Initial target for simulated digging was set at 3 sets of 8 repetitions. | Participants were progressed upon successful completion on the initial exercise. Kneeling was progressed from light application of pressure over the anterior knee in sitting to unilateral kneeling on to cushioned chair in standing. Duration of kneeling exercise was progressed according to participant tolerance up to a maximum of 60 seconds. Progression to high kneeling on a cushioned chair, high kneeling on a soft surface on the floor with further increase in knee flexion as tolerated. Simulated digging was progressed by increasing the number of sets and repetitions and increasing the resistance of the stepper machine. |
| Lunges | Participants performed standing lunges. Initial target was 2 sets of 8 repetitions of shallow lunges | Participants were progressed upon successful completion on the initial exercise. Progressions included increasing sets and repetitions, depth of lunge, reduced support, lunge walking, lunging to pick up an object from the floor and bowling. |
| Treadmill  Cross-trainer | Walking on a treadmill or exercising on a cross-trainer. Initial target was set at 1 minute | Progressions for the treadmill exercise included increasing duration, speed, incline (treadmill only) and resistance (treadmill only). |

# Supplementary material 6: Class size effect on the LEFS at 12 months post-operative

The categorical variable used as the treatment group variable before was recoded as follows:

1. Participant in the implementer group who did not attend any classes
2. Participant in the implementer group who has a median class size of 1 to 3 patients
3. Participant in the implementer group who has a median class size of more than 3 patients;

|  |  |  |  | Adjusted for stratification variables | | | Adjusted for stratification variables and baseline imbalance^3,4^ | | |
| --- | --- | --- | --- | --- | --- | --- | --- | --- | --- |
|  | N | Mean (SD) | 95% CI | Difference in means ^1,4^ | 95% CI | p-value | Difference in means^1,4^ | 95% CI | p-value |
| Usual Care group^2^ | 83 | 53.29 (17.53) | (49.46, 57.12) |  |  | 0.091 |  |  | 0.106 |
| Intervention group (1) | 11 | 47.91 (17.51) | (36.15, 59.67) | -4.77 | (-14.68, 5.14) |  | -4.84 | (-14.36, 4.68) |  |
| Intervention group (2) | 13 | 61 (16.96) | (50.75, 71.25) | 5.04 | (-4.25, 14.33) |  | 5.43 | (-3.77, 14.63) |  |
| Intervention group (3) | 57 | 56.12 (18.79) | (51.14, 61.11) | 5.43 | (0.07, 10.80) |  | 5.11 | (-0.17, 10.40) |  |

^1^ In comparison to reference group

^2^ Reference group

^3^Variables which were imbalanced at baseline: Level of education; working status; pre-operative HADS anxiety

^4^Using linear regression with a categorical exposure

# Supplementary material 7: Summary of all outcomes by arm and timepoint

| Outcome measures |  | 3months | | 6 months | | 12 months | |
| --- | --- | --- | --- | --- | --- | --- | --- |
|  |  | n |  | n |  | n |  |
| LEFS  mean (SD) | Intervention | 80 | 51 (14) | 79 | 55 (16) | 81 | 56 (19) |
|  | Usual Care | 76 | 45 (15) | 73 | 52 (17) | 83 | 53 (18) |
| KOOS Pain  mean (SD) | Intervention | 67 | 72 (18) | 66 | 79 (20) | 66 | 83 (20) |
|  | Usual Care | 62 | 70 (20) | 61 | 79 (19) | 67 | 81 (21) |
| KOOS Symptoms  mean (SD) | Intervention | 74 | 65 (17) | 71 | 73 (18) | 74 | 77 (17) |
|  | Usual Care | 67 | 66 (17) | 67 | 74 (19) | 67 | 77 (20) |
| KOOS ADL  mean (SD) | Intervention | 63 | 77 (17) | 58 | 80 (19) | 66 | 82 (17) |
|  | Usual Care | 60 | 73 (19) | 62 | 79 (19) | 57 | 81 (20) |
| KOOS Sport/Rec  median (IQR) | Intervention | 71 | 30 (15, 50) | 63 | 35 (20, 65) | 61 | 45 (25, 75) |
|  | Usual Care | 58 | 25 (5, 40) | 60 | 45 (15, 65) | 55 | 45 (25, 65) |
| KOOS QOL  median (IQR) | Intervention | 75 | 56 (44, 75) | 71 | 63 (50, 81) | 72 | 75 (50, 94) |
|  | Usual Care | 68 | 56 (44, 69) | 65 | 69 (50, 75) | 69 | 63 (56, 88) |
| KOOS Total  mean (SD) | Intervention | 53 | 62 (15) | 49 | 68 (20) | 54 | 73 (19) |
|  | Usual Care | 49 | 60 (17) | 54 | 67 (19) | 48 | 68 (21) |
| HADS: Anxiety  median (IQR) | Intervention | 76 | 3 (2, 6) | 72 | 4 (1, 7) | 73 | 3 (1, 7) |
|  | Usual Care | 68 | 3 (1, 7) | 66 | 2 (0, 4) | 68 | 3 (1, 6) |
| HADS: Depression  median (IQR) | Intervention | 76 | 3 (1, 6) | 72 | 3 (1, 6) | 73 | 2 (1, 5) |
|  | Usual Care | 68 | 2 (1, 5) | 66 | 2 (1, 5) | 69 | 2 (1, 5) |
| Satisfaction with surgery  median (IQR) | Intervention | 56 | 94 (84, 100) | 58 | 100 (81, 100) | 65 | 100 (88, 100) |
|  | Usual Care | 51 | 94 (81, 100) | 56 | 100 (88, 100) | 61 | 100 (81, 100) |

| Outcome measures |  | Category | 3months | | 6 months | | 12 months | |
| --- | --- | --- | --- | --- | --- | --- | --- | --- |
|  |  |  | N | n (%) | N | n (%) | N | n (%) |
| Satisfaction with physiotherapy | Intervention | Very satisfied | 74 | 51 (69) | 71 | 46 (65) | 70 | 50 (71) |
|  |  | Somewhat satisfied |  | 11 (15) |  | 11 (15) |  | 10 (14) |
|  |  | Neither satisfied or dissatisfied |  | 6 (8) |  | 9 (13) |  | 4 (6) |
|  |  | Somewhat dissatisfied |  | 1 (1) |  | 2 (3) |  | 1 (1) |
|  |  | Very dissatisfied |  | 5 (7) |  | 3 (4) |  | 5 (7) |
|  | Usual Care | Very satisfied | 65 | 25 (38) | 64 | 26 (41) | 67 | 29 (43) |
|  |  | Somewhat satisfied |  | 8 (12) |  | 12 (19) |  | 9 (13) |
|  |  | Neither satisfied or dissatisfied |  | 15 (23) |  | 16 (25) |  | 14 (21) |
|  |  | Somewhat dissatisfied |  | 7 (11) |  | 3 (5) |  | 11 (16) |
|  |  | Very dissatisfied |  | 10 (15) |  | 7 (11) |  | 4 (6) |

# Supplementary material 8: Histogram of LEFS at 12 months by trial arm


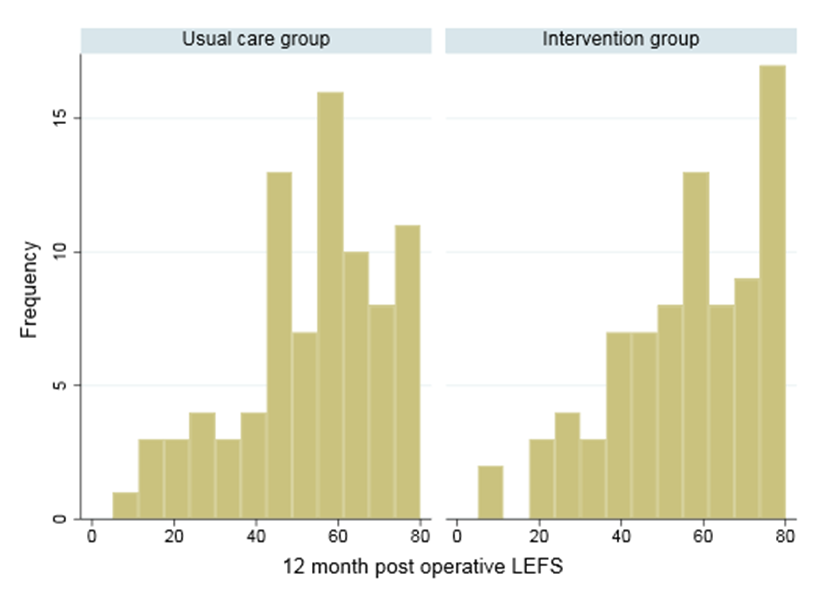


# Supplementary material 9: Primary analysis^1^ of LEFS at 12 months – additional missing data analyses

Modelling strategy for analysis of LEFS:

- Model 1: linear mixed regression adjusted for stratification variables and accounting for clustering within patient.
- Model 2: linear mixed regression adjusted for stratification variables and accounting for clustering within patient and surgeon.
- Model 3: linear mixed regression adjusted for stratification variables and baseline imbalance variables (Level of education; working status; HADS anxiety) and accounting for clustering within patient and surgeon.
- Model 4: linear mixed regression adjusted for stratification variables and whether the patient had received additional physiotherapy during the trial and accounting for clustering within patient and surgeon.

| Adjustments | N paritcipants^4^ Intervention | N participants^4^ Usual Care | Difference in means | 95% CI | p-value |
| --- | --- | --- | --- | --- | --- |
| Analysis using the 10% worse approach for missing data (intention-to-treat)^5^ | | | | | |
| Model 1 | 89 | 91 | 3.70 | (-0.24, 7.63) | 0.066 |
| Model 2^2^ | 89 | 91 | 3.70 | (-0.24, 7.63) | 0.066 |
| Model 3^3^ | 89 | 91 | 3.61 | (-0.27, 7.50) | 0.068 |
| Model 4 | 89 | 91 | 3.18 | (-0.83, 7.20) | 0.120 |
| Analysis using the 10% better approach for missing data (intention-to-treat)^6^ | | | | | |
| Model 1 | 89 | 91 | 3.68 | (-0.33, 7.69) | 0.072 |
| Model 2^2^ | 89 | 91 | 3.68 | (-0.33, 7.69) | 0.072 |
| Model 3^3^ | 89 | 91 | 3.62 | (-0.31, 7.55) | 0.071 |
| Model 4 | 89 | 91 | 3.42 | (-0.64, 7.47) | 0.099 |

^1^ Linear mixed model adjusted for repeated measurements

^2^ The variance of the random effect associated with surgeon level was significant; this level was kept for the following sensitivity analyses

^3^ Variables which were imbalanced at baseline: Level of education; working status; pre-operative HADS anxiety

^4^ Number of participants contributing data for the analysis

^5^ Missing values of the outcome were imputed using a value 10% less than the mean of the complete outcomes

^6^ Missing values of the outcome were imputed using a value 10% more than the mean of the complete outcomes

# Supplementary material 10: Investigation of gender-specific effect of the intervention effect

A linear mixed regression on LEFS at 12 months adjusted for stratification variables, gender and interaction between the interview effect terms and gender and accounting for clustering within patient and surgeon was used for this analysis.

|  | Gender | Difference in means (treatment effect) | 95% CI | Difference in treatment effects (Interaction coefficient) | 95% CI | P-value |
| --- | --- | --- | --- | --- | --- | --- |
| ITT | Male | 6.88 | (0.97, 12.79) | -4.55 | (-12.17, 3.07) | 0.242 |
|  | Female | 2.33 | (-3.19, 7.84) |  |  |  |
| MICE | Male | 6.82 | (0.80, 12.85) | -4.75 | (-12.42, 2.93) | 0.225 |
|  | Female | 2.08 | (-3.48, 7.64) |  |  |  |
| +10% | Male | 5.98 | (0.36, 11.61) | -3.74 | (-10.85, 3.36) | 0.302 |
|  | Female | 2.24 | (-3.02, 7.50) |  |  |  |
| -10% | Male | 5.54 | (-0.01, 11.09) | -3.54 | (-10.55, 3.47) | 0.322 |
|  | Female | 2.00 | (-3.19, 7.18) |  |  |  |
| Per-protocol | Male | 8.45 | (2.20, 14.70) | -4.40 | (-12.45, 3.66) | 0.285 |
|  | Female | 4.05 | (-1.80, 9.90) |  |  |  |


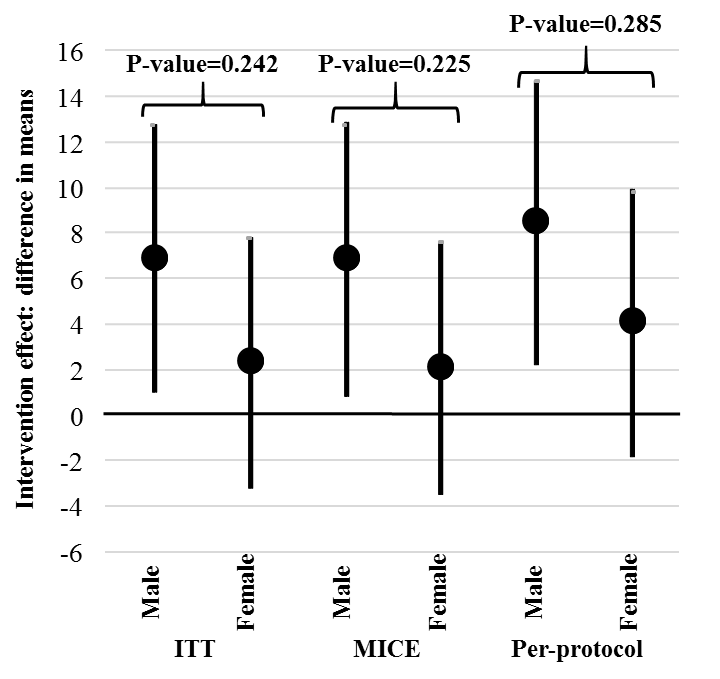


**Intervention effect by gender**

# Supplementary material 11.1: LEFS at 3 months: Sensitivity and per protocol analyses

**Adjusted analysis^1^**

|  | Intervention | Usual Care |  |  |  |
| --- | --- | --- | --- | --- | --- |
|  | N Participants ^4^ | N Participants ^4^ | Difference in means | 95% CI | P-value |
| Model 2^2^ | 87 | 86 | 8.04 | (3.73, 12.36) | <0.001 |
| Model 3^3^ | 87 | 86 | 7.86 | (3.63, 12.08) | <0.001 |
| Model 4 | 87 | 86 | 7.49 | (3.21, 11.76) | 0.001 |

^1^ Linear mixed model using appropriate contrast effect to assess treatment effect at 3 months on LEFS, adjusted for repeated measurements

^2^ The variance of the random effect associated with surgeon level was significant, this level will be kept for the following sensitivity analyses

^3^ Variables which were imbalanced at baseline: Level of education; working status; pre-operative HADS anxiety

^4^ Number of participants contributing data for the analysis

**Analysis accounting for missing data**

|  |  | Intervention | Usual Care |  |  |  |
| --- | --- | --- | --- | --- | --- | --- |
|  | Adjustment | N Participants | N Participants | Difference in means | 95% CI | P-value |
| Analysis using MICE to account for missing data | Model 1 | 89 | 91 | 7.59 | (3.22, 11.96) | 0.001 |
|  | Model 2 | 89 | 91 | 7.57 | (3.20, 11.94) | 0.001 |
|  | Model 3 | 89 | 91 | 7.97 | (3.68, 12.26) | <0.001 |
|  | Model 4 | 89 | 91 | 7.13 | (2.77, 11.48) | 0.001 |
| Analysis using the 10% worse approach^1^ | Model 1 | 89 | 91 | 7.35 | (3.42, 11.28) | <0.001 |
|  | Model 2 | 89 | 91 | 7.35 | (3.42, 11.28) | <0.001 |
|  | Model 3 | 89 | 91 | 7.27 | (3.38, 11.15) | <0.001 |
|  | Model 4 | 89 | 91 | 7.02 | (3.00, 11.03) | 0.001 |
| Analysis using the 10% better approach^2^ | Model 1 | 89 | 91 | 6.7 | (2.68, 10.71) | 0.001 |
|  | Model 2 | 89 | 91 | 6.69 | (2.68, 10.71) | 0.001 |
|  | Model 3 | 89 | 91 | 6.63 | (2.70, 10.56) | 0.001 |
|  | Model 4 | 89 | 91 | 6.53 | (2.47, 10.58) | 0.002 |

^1^ Missing values of the outcome were imputed using a value 10% less than the mean of the complete outcomes

^2^ Missing values of the outcome were imputed using a value 10% more than the mean of the complete outcomes

**Per protocol analysis**

|  |  | Intervention | Usual Care |  |  |  |
| --- | --- | --- | --- | --- | --- | --- |
|  | Adjustment | N Participants | N Participants | Difference in means | 95% CI | P-value |
| Analysis using the Per Protocol principle | Model 1 | 69 | 86 | 8.45 | (3.88, 13.03) | <0.001 |
|  | Model 2 | 69 | 86 | 8.44 | (3.87, 13.01) | <0.001 |
|  | Model 3 | 69 | 86 | 8.67 | (4.15, 13.19) | <0.001 |
|  | Model 4 | 69 | 86 | 7.76 | (3.28, 12.24) | 0.001 |

# Supplementary material 11.2: LEFS at 6 months: Sensitivity and per protocol analyses

**Adjusted analysis1**

|  | Intervention | Usual Care |  |  |  |
| --- | --- | --- | --- | --- | --- |
|  | N Participants^4^ | N Participants^4^ | Difference in means | 95% CI | P-value |
| Model 2^2^ | 87 | 86 | 5.38 | (1.03, 9.73) | 0.015 |
| Model 3^3^ | 87 | 86 | 5.13 | (0.87, 9.39) | 0.018 |
| Model 4 | 87 | 86 | 4.9 | (0.60, 9.20) | 0.025 |

^1^ Linear mixed model using appropriate contrast effect to assess treatment effect at 6 months on LEFS, adjusted for repeated measurements

^2^ The variance of the random effect associated with surgeon level was significant, this level will be kept for the following sensitivity analyses

^3^ Variables which were imbalanced at baseline: Level of education; working status; pre-operative HADS anxiety

^4^ Number of participants contributing data for the analysis

**Analysis accounting for missing data**

|  |  | Intervention | Usual Care |  |  |  |
| --- | --- | --- | --- | --- | --- | --- |
|  | Adjustment | N Participants | N Participants | Difference in means | 95% CI | P-value |
| Analysis using MICE to account for missing data | Model 1 | 89 | 91 | 5.09 | (0.59, 9.60) | 0.027 |
|  | Model 2 | 89 | 91 | 5.07 | (0.56, 9.58) | 0.027 |
|  | Model 3 | 89 | 91 | 5.34 | (0.87, 9.80) | 0.019 |
|  | Model 4 | 89 | 91 | 4.51 | (0.02, 9.00) | 0.049 |
| Analysis using the 10% worse approach^1^ | Model 1 | 89 | 91 | 4.11 | (0.17, 8.04) | 0.041 |
|  | Model 2 | 89 | 91 | 4.11 | (0.17, 8.04) | 0.041 |
|  | Model 3 | 89 | 91 | 4.02 | (0.14, 7.91) | 0.042 |
|  | Model 4 | 89 | 91 | 3.63 | (-0.39, 7.65) | 0.077 |
| Analysis using the 10% better approach^2^ | Model 1 | 89 | 91 | 3.42 | (-0.71, 7.55) | 0.105 |
|  | Model 2 | 89 | 91 | 3.42 | (-0.71, 7.55) | 0.105 |
|  | Model 3 | 89 | 91 | 3.09 | (-0.84, 7.02) | 0.123 |
|  | Model 4 | 89 | 91 | 2.85 | (-1.20, 6.91) | 0.168 |

^1^ Missing values of the outcome were imputed using a value 10% less than the mean of the complete outcomes

^2^ Missing values of the outcome were imputed using a value 10% more than the mean of the complete outcomes

**Per protocol analysis**

|  |  | Intervention | Usual Care |  |  |  |
| --- | --- | --- | --- | --- | --- | --- |
|  | Adjustment | N Participants | N Participants | Difference in means | 95% CI | P-value |
| Analysis using the Per Protocol principle | Model 1 | 69 | 86 | 5.91 | (1.33, 10.5) | 0.012 |
|  | Model 2 | 69 | 86 | 5.9 | (1.31, 10.48) | 0.012 |
|  | Model 3 | 69 | 86 | 6.04 | (1.51, 10.58) | 0.009 |
|  | Model 4 | 69 | 86 | 5.27 | (0.78, 9.76) | 0.021 |

**Change in treatment effect on LEFS between 3, 6 and 12 months**

| Treatment effect (TE) on LEFS^1^ | Coefficient | 95% CI | p-value |
| --- | --- | --- | --- |
| How 3 month TE differs from 12 month TE | 3.60^2^ | (0.06, 7.15) | 0.046 |
| How 6 month TE differs from 12 month TE | 0.94^3^ | (-2.62, 4.50) | 0.603 |
| How 3 month TE differs from 6 month TE | 2.66^4^ | (-0.95, 6.27) | 0.149 |

^1^Derived using appropriate contrasts, i.e difference of linear combinations of parameters derived from Model 1

^2^The 3 month treatment effect is 3.60 LEFS points higher that the treatment effect at 12 months

^3^The 6 month treatment effect is 0.94 LEFS points higher that the treatment effect at 12 month

^4^The 3 month treatment effect is 2.66 LEFS points higher that the treatment effect at 6 months

# Supplementary material 11.3: KOOS Total: Sensitivity and per protocol analyses

The KOOS is a secondary outcome. It consists of 5 subscales; pain, other symptoms, function in daily living (ADL), function in sport and recreation (Sport/Rec) and knee related quality of life (QOL). Standardized answer options are given (5 Likert boxes) and each item is assigned a score from 0 (none) to 4 (extreme). A normalized score (100 indicating no symptoms and 0 indicating extreme symptoms)

Modelling strategy for analysis of KOOS total and subscales:

- Model 1: Ordinal logistic mixed regression adjusted for stratification variables, pre-operative KOOS score and accounting for clustering within patient.
- Model 2: Ordinal logistic mixed regression adjusted for stratification variables, pre-operative KOOS score and accounting for clustering within patient and surgeon.
- Model 3: Ordinal logistic mixed regression adjusted for stratification variables, pre-operative KOOS score and baseline imbalance variables (Level of education; working status; HADS anxiety) and accounting for clustering within patient.
- Model 4: Ordinal logistic mixed regression adjusted for stratification variables, pre-operative KOOS score and whether the patient had received additional physiotherapy during the trial and accounting for clustering within patient.

**Adjusted analysis1**

|  | N Intervention | N Usual Care | Odds Ratio | 95% CI | P-value |
| --- | --- | --- | --- | --- | --- |
| Model 2^2, 3^ | 75 | 71 | 2.53 | (0.51, 12.46) | 0.255 |
| Model 3 | 75 | 71 | 2.36 | (0.49, 11.28) | 0.283 |
| Model 4 | 75 | 71 | 2.14 | (0.44, 10.54) | 0.348 |

^1^ Ordinal logistic mixed model for averaged KOOS adjusted for repeated measurements (two-level model: measurement/patient)

^2^ Convergence not achieved in three-level mixed model(measurement/patient/surgeon) – surgeon level included as a covariate in two-level mixed model

^3^Surgeon variable was not significant in Model 2 and so was not included in Model 3 and Model 4

**Analysis accounting for missing data**

|  | Adjustment | N Intervention | N Usual Care | Odds Ratio | 95% CI | P-value |
| --- | --- | --- | --- | --- | --- | --- |
| Analysis using MICE to account for missing data^1^ | Model 1 | 89 | 91 | 1.38 | (0.66, 2.87) | 0.384 |
|  | Model 2 | 89 | 91 | 1.37 | (0.66, 2.86) | 0.388 |
|  | Model 3 | 89 | 91 | 1.55 | (0.74, 3.24) | 0.243 |
|  | Model 4 | 89 | 91 | 1.36 | (0.66, 2.80) | 0.405 |
| Analysis using the 10% worse approach^2^ | Model 1 | 89 | 91 | 1.91 | (0.72, 5.07) | 0.195 |
|  | Model 2 | 89 | 91 | 1.91 | (0.72, 5.07) | 0.195 |
|  | Model 3 | 89 | 91 | 1.98 | (0.75, 5.22) | 0.168 |
|  | Model 4 | 89 | 91 | 1.73 | (0.65, 4.60) | 0.270 |
| Analysis using the 10% better approach^3^ | Model 1 | 89 | 91 | 1.00 | (0.43, 2.25) | 0.979 |
|  | Model 2 | 89 | 91 | 1.00 | (0.43, 2.25) | 0.979 |
|  | Model 3 | 89 | 91 | 1.06 | (0.47, 2.39) | 0.886 |
|  | Model 4 | 89 | 91 | 1.03 | (0.46, 2.33) | 0.939 |

**^1^** MICE only: Not multi-level models, single-level ordinal logistic models used for MICE analysis only

^2^ Missing observations are replaced by category 3 (51 ≤ KOOS total ≤ 75)

^3^ Missing observations are replaced by category 4 (KOOS total >75)

|  | Adjustment | N Intervention | N Usual Care | Odds Ratio | 95% CI | P-value |
| --- | --- | --- | --- | --- | --- | --- |
| Analysis using the Per Protocol principle | Model 1 | 61 | 71 | 2.57 | (0.49, 13.44) | 0.263 |
|  | Model 2 | 61 | 71 | 2.55 | (0.49, 13.31) | 0.268 |
|  | Model 3 | 61 | 71 | 2.55 | (0.50, 13.06) | 0.261 |
|  | Model 4 | 61 | 71 | 2.06 | (0.41, 10.65) | 0.388 |

**Per protocol analysis**

**Change in treatment effect on KOOS total between 3, 6 and 12 months**

| Treatment effect (TE) ^1^ | Odds ratio | 95% CI | p-value |
| --- | --- | --- | --- |
| How 3 month TE differs from 12 month TE | 1.13 | (0.24, 5.35) | 0.876 |
| How 6 month TE differs from 12 month TE | 0.98 | (0.21, 4.70) | 0.982 |
| How 3 month TE differs from 6 month TE | 0.87 | (0.18, 4.11) | 0.858 |

^1^Derived using contrasts, i.e. difference of appropriate parameters combination assessed in Model 1

# Supplementary material 11.4: KOOS QOL: Sensitivity and per protocol analyses

**Adjusted analysis1**

|  | N Intervention | N Usual Care | Odds Ratio | 95% CI | P-value |
| --- | --- | --- | --- | --- | --- |
| Model 2^2^ | 84 | 77 | 1.82 | (0.62, 5.32) | 0.272 |
| Model 3^3^ | 84 | 77 | 2.15 | (0.74, 6.25) | 0.159 |
| Model 4 | 84 | 77 | 1.58 | (0.55, 4.55) | 0.398 |

^1^ Ordinal Logistic mixed model for averaged KOOS QoL adjusted for repeated measurements (two-level model: measurement/patient)

^2^ Convergence not achieved in three-level mixed model(measurement/patient/surgeon) – surgeon level included as a covariate in two-level mixed model

^3^ Variables which were imbalanced at baseline: Level of education; working status; pre-operative HADS anxiety

Analysis accounting for missing data

|  | Adjustment | N Intervention | N Usual Care | Odds Ratio | 95% CI | P-value |
| --- | --- | --- | --- | --- | --- | --- |
| Analysis using MICE to account for missing data | Model 1 | 89 | 91 | 1.46 | (0.78, 2.74) | 0.231 |
|  | Model 2 | 89 | 91 | 1.47 | (0.78, 2.74) | 0.23 |
|  | Model 3 | 89 | 91 | 1.69 | (0.89, 3.23) | 0.109 |
|  | Model 4 | 89 | 91 | 1.41 | (0.74, 2.70) | 0.295 |
| Analysis using the 10% worse approach^2^ | Model 1 | 89 | 91 | 1.58 | (0.68, 3.72) | 0.290 |
|  | Model 2 | 89 | 91 | 1.58 | (0.68, 3.72) | 0.290 |
|  | Model 3 | 89 | 91 | 1.79 | (0.77, 4.16) | 0.179 |
|  | Model 4 | 89 | 91 | 1.47 | (0.64, 3.41) | 0.364 |
| Analysis using the 10% better approach^3^ | Model 1 | 89 | 91 | 1.16 | (0.47, 2.89) | 0.743 |
|  | Model 2 | 89 | 91 | 1.17 | (0.47, 2.90) | 0.741 |
|  | Model 3 | 89 | 91 | 1.28 | (0.52, 3.15) | 0.588 |
|  | Model 4 | 89 | 91 | 1.03 | (0.43, 2.50) | 0.942 |

**^1^** Not multi-level models, single-level ordinal logistic models used for MICE analysis only.

^2^ Missing observations are replaced by category 3 (51 ≤ KOOS QOL ≤ 75)

^3^ Missing observations are replaced by category 4 (KOOS QOL >75)

**Per protocol analysis**

|  | Adjustment | N Intervention | N Usual Care | Odds Ratio | 95% CI | P-value |
| --- | --- | --- | --- | --- | --- | --- |
| Analysis using the Per Protocol principle | Model 1 | 68 | 77 | 1.97 | (0.63, 6.16) | 0.244 |
|  | Model 2 | 68 | 77 | 1.97 | (0.63, 6.15) | 0.245 |
|  | Model 3 | 68 | 77 | 2.70 | (0.85, 8.52) | 0.091 |
|  | Model 4 | 68 | 77 | 1.65 | (0.53, 5.09) | 0.384 |

**Change in treatment effect on KOOS QOL between 3, 6 and 12 months**

| Treatment effect (TE) ^1^ | Odds ratio | 95% CI | p-value |
| --- | --- | --- | --- |
| How 3 month TE differs from 12 month TE | 0.57 | (0.20, 1.59) | 0.283 |
| How 6 month TE differs from 12 month TE | 1.30 | (0.45, 3.68) | 0.636 |
| How 3 month TE differs from 6 month TE | 2.26 | (0.81, 6.28) | 0.118 |

^1^Derived using contrasts, i.e. difference of appropriate parameters combination assessed in Model 1

# Supplementary material 11.5: KOOS ADL: Sensitivity and per protocol analyses

**Adjusted analysis^1^**

|  | N Intervention | N Usual Care | Odds Ratio | 95% CI | P-value |
| --- | --- | --- | --- | --- | --- |
| Model 2^2^ | 80 | 75 | 2.17 | (0.51, 9.14) | 0.291 |
| Model 3^3^ | 80 | 75 | 2.12 | (0.51, 8.87) | 0.305 |
| Model 4 | 80 | 75 | 1.73 | (0.42, 7.16) | 0.451 |

^1^Ordinal logistic mixed model for KOOS ADL adjusted for repeated measurements (two-level model: measurement/patient)

^2^Convergence not achieved in three-level mixed model(measurement/patient/surgeon) – surgeon level included as a covariate in two-level mixed model ^3^Variables which were imbalanced at baseline: Level of education; working status; pre-operative HADS anxiety

Analysis accounting for missing data

|  | Adjustment | N Intervention | N Usual Care | Odds Ratio | 95% CI | P-value |
| --- | --- | --- | --- | --- | --- | --- |
| Analysis using MICE to account for missing data^1^ | Model 1 | 89 | 91 | 1.16 | (0.52, 2.59) | 0.720 |
|  | Model 2 | 89 | 91 | 1.17 | (0.52, 2.63) | 0.697 |
|  | Model 3 | 89 | 91 | 1.17 | (0.52, 2.67) | 0.697 |
|  | Model 4 | 89 | 91 | 1.12 | (0.50, 2.51) | 0.784 |
| Analysis using the 10% worse approach^2^ | Model 1 | 89 | 91 | 1.70 | (0.66, 4.44) | 0.274 |
|  | Model 2 | 89 | 91 | 1.70 | (0.66, 4.44) | 0.274 |
|  | Model 3 | 89 | 91 | 1.81 | (0.69, 4.72) | 0.225 |
|  | Model 4 | 89 | 91 | 1.64 | (0.63, 4.27) | 0.308 |
| Analysis using the 10% better approach^3^ | Model 1 | 89 | 91 | 0.74 | (0.28, 1.98) | 0.553 |
|  | Model 2 | 89 | 91 | 0.74 | (0.28, 1.98) | 0.554 |
|  | Model 3 | 89 | 91 | 0.75 | (0.28, 1.98) | 0.559 |
|  | Model 4 | 89 | 91 | 0.65 | (0.25, 1.70) | 0.380 |

**^1^** Not multi-level models, single-level ordinal logistic models used for MICE analysis only.

^2^ Missing observations are replaced by category 3 (51 ≤ KOOS ADL ≤ 75)

^3^ Missing observations are replaced by category 4 (KOOS ADL >75)

Per protocol analysis

|  | Adjustment | N Intervention | N Usual Care | Odds Ratio | 95% CI | P-value |
| --- | --- | --- | --- | --- | --- | --- |
| Analysis using the Per Protocol principle | Model 1 | 65 | 75 | 1.49 | (0.35, 6.35) | 0.593 |
|  | Model 2 | 65 | 75 | 1.49 | (0.35, 6.35) | 0.593 |
|  | Model 3 | 65 | 75 | 1.51 | (0.35, 6.49) | 0.578 |
|  | Model 4 | 65 | 75 | 1.13 | (0.27, 4.75) | 0.868 |

**Change in treatment effect on KOOS ADL between 3, 6 and 12 months**

| Treatment effect (TE) ^1^ | Odds ratio | 95% CI | p-value |
| --- | --- | --- | --- |
| How 3 month TE differs from 12 month TE | 2.48 | (0.57, 10.73) | 0.224 |
| How 6 month TE differs from 12 month TE | 1.48 | (0.33, 6.67) | 0.606 |
| How 3 month TE differs from 6 month TE | 0.60 | (0.14, 2.55) | 0.488 |

^1^Derived using contrasts, i.e. difference of appropriate parameters combination assessed in Model 1

# Supplementary material 11.6: KOOS Pain: Sensitivity and per protocol analyses

**Adjusted analysis^1^**

|  | N Intervention | N Usual Care | Odds Ratio | 95% CI | P-value |
| --- | --- | --- | --- | --- | --- |
| Model 2^2^ | 84 | 76 | 2.80 | (0.78, 10.00) | 0.114 |
| Model 3^3^ | 84 | 76 | 2.79 | (0.79, 9.86) | 0.112 |
| Model 4 | 84 | 76 | 2.58 | (0.72, 9.25) | 0.147 |

^1^Ordinal logistic mixed model for KOOS pain adjusted for repeated measurements (two-level model: measurement/patient)

^2^ Convergence not achieved in three-level mixed model(measurement/patient/surgeon) – surgeon level included as a covariate in two-level mixed model

^3^ Variables which were imbalanced at baseline: Level of education; working status; pre-operative HADS anxiety

**Analysis accounting for missing data**

|  | Adjustment | N Intervention | N Usual Care | Odds Ratio | 95% CI | P-value |
| --- | --- | --- | --- | --- | --- | --- |
| Analysis using MICE to account for missing data | Model 1 | 89 | 91 | 1.57 | (0.83, 2.97) | 0.169 |
|  | Model 2 | 89 | 91 | 1.57 | (0.83, 2.97) | 0.167 |
|  | Model 3 | 89 | 91 | 1.75 | (0.90, 3.41) | 0.100 |
|  | Model 4 | 89 | 91 | 1.53 | (0.80, 2.92) | 0.193 |
| Analysis using the 10% worse approach^2^ | Model 1 | 89 | 91 | 1.77 | (0.73, 4.33) | 0.209 |
|  | Model 2 | 89 | 91 | 1.77 | (0.73, 4.33) | 0.209 |
|  | Model 3 | 89 | 91 | 1.84 | (0.75, 4.47) | 0.180 |
|  | Model 4 | 89 | 91 | 1.76 | (0.72, 4.29) | 0.217 |
| Analysis using the 10% better approach^3^ | Model 1 | 89 | 91 | 1.55 | (0.60, 3.97) | 0.364 |
|  | Model 2 | 89 | 91 | 1.55 | (0.60, 3.97) | 0.364 |
|  | Model 3 | 89 | 91 | 1.61 | (0.63, 4.07) | 0.317 |
|  | Model 4 | 89 | 91 | 1.45 | (0.57, 3.71) | 0.435 |

**^1^** Not multi-level models, single-level ordinal logistic models used for MICE analysis only.

^2^ Missing observations are replaced by category 3 (51 ≤ KOOS pain ≤ 75)

^3^ Missing observations are replaced by category 4 (KOOS pain >75)

|  | Adjustment | N Intervention | N Usual Care | Odds Ratio | 95% CI | P-value |
| --- | --- | --- | --- | --- | --- | --- |
| Analysis using the Per Protocol principle | Model 1 | 68 | 76 | 3.07 | (0.78, 12.02) | 0.107 |
|  | Model 2 | 68 | 76 | 3.07 | (0.78, 12.01) | 0.107 |
|  | Model 3 | 68 | 76 | 3.34 | (0.86, 13.04) | 0.082 |
|  | Model 4 | 68 | 76 | 2.76 | (0.70, 10.85) | 0.147 |

**Per protocol analysis**

**Change in treatment effect on KOOS pain between 3, 6 and 12 months**

| Treatment effect (TE)^1^ | Odds ratio | 95% CI | p-value |
| --- | --- | --- | --- |
| How 3 month TE differs from 12 month TE | 0.79 | (0.22, 2.84) | 0.721 |
| How 6 month TE differs from 12 month TE | 0.58 | (0.16, 2.15) | 0.417 |
| How 3 month TE differs from 6 month TE | 0.73 | (0.21, 2.53) | 0.625 |

^1^Derived using contrasts, i.e. difference of appropriate parameters combination assessed in Model 1

# Supplementary material 11.7: KOOS Sport/Rec: Sensitivity and per protocol analyses

**Adjusted analysis^1^**

|  | N Intervention | N Usual Care | Odds Ratio | 95% CI | P-value |
| --- | --- | --- | --- | --- | --- |
| Model 2^2^ | 82 | 74 | 2.30 | (0.64, 8.19) | 0.199 |
| Model 3^3^ | 82 | 74 | 1.93 | (0.54, 6.88) | 0.311 |
| Model 4 | 82 | 74 | 1.93 | (0.55, 6.79) | 0.305 |

^1^Ordinal logistic mixed model for KOOS sport/rec adjusted for repeated measurements (two-level model: measurement/patient)

^2^ Convergence not achieved in three-level mixed model(measurement/patient/surgeon) – surgeon level included as a covariate in two-level mixed model

^3^ Variables which were imbalanced at baseline: Level of education; Working status; pre-operative HADS anxiety

Analysis accounting for missing data

|  | Adjustment | N Intervention | N Usual Care | Odds Ratio | 95% CI | P-value |
| --- | --- | --- | --- | --- | --- | --- |
| Analysis using MICE to account for missing data | Model 1 | 89 | 91 | 1.38 | (0.72, 2.64) | 0.337 |
|  | Model 2 | 89 | 91 | 1.38 | (0.71, 2.65) | 0.339 |
|  | Model 3 | 89 | 91 | 1.47 | (0.73, 2.92) | 0.276 |
|  | Model 4 | 89 | 91 | 1.32 | (0.67, 2.57) | 0.417 |
| Analysis using the 10% worse approach^2^ | Model 1 | 89 | 91 | 1.49 | (0.61, 3.68) | 0.384 |
|  | Model 2 | 89 | 91 | 1.49 | (0.61, 3.68) | 0.384 |
|  | Model 3 | 89 | 91 | 1.45 | (0.59, 3.57) | 0.416 |
|  | Model 4 | 89 | 91 | 1.39 | (0.57, 3.36) | 0.471 |
| Analysis using the 10% better approach^3^ | Model 1 | 89 | 91 | 0.98 | (0.42, 2.27) | 0.960 |
|  | Model 2 | 89 | 91 | 0.98 | (0.42, 2.27) | 0.961 |
|  | Model 3 | 89 | 91 | 1.01 | (0.44, 2.34) | 0.979 |
|  | Model 4 | 89 | 91 | 0.90 | (0.39, 2.07) | 0.810 |

^1^ Not multi-level models, single-level ordinal logistic models used for MICE analysis only.

^2^ Missing observations are replaced by category 3 (51 ≤ KOOS sport ≤ 75)

^3^ Missing observations are replaced by category 4 (KOOS sport >75)

Per protocol analysis

|  | Adjustment | N Intervention | N Usual Care | Odds Ratio | 95% CI | P-value |
| --- | --- | --- | --- | --- | --- | --- |
| Analysis using the Per Protocol principle | Model 1 | 67 | 74 | 3.23 | (0.81, 12.77) | 0.095 |
|  | Model 2 | 67 | 74 | 3.21 | (0.82, 12.61) | 0.095 |
|  | Model 3 | 67 | 74 | 2.77 | (0.68, 11.19) | 0.153 |
|  | Model 4 | 67 | 74 | 2.57 | (0.66, 9.97) | 0.171 |

**Change in treatment effect on KOOS Sport/Rec between 3, 6 and 12 months**

| Treatment effect (TE)^1^ | Odds ratio | 95% CI | p-value |
| --- | --- | --- | --- |
| How 3 month TE differs from 12 month TE | 0.99 | (0.29, 3.38) | 0.985 |
| How 6 month TE differs from 12 month TE | 0.93 | (0.29, 3.04) | 0.908 |
| How 3 month TE differs from 6 month TE | 0.94 | (0.28, 3.13) | 0.925 |

^1^Derived using contrasts, i.e. difference of appropriate parameters combination assessed in Model 1

# Supplementary material 11.8: KOOS Symptoms: Sensitivity and per protocol analyses

**Adjusted analysis^1^**

|  | N Intervention | N Usual Care | Odds Ratio | 95% CI | P-value |
| --- | --- | --- | --- | --- | --- |
| Model 2^2^ | 85 | 77 | 1.77 | (0.49, 6.43) | 0.386 |
| Model 3^3^ | 85 | 77 | 1.88 | (0.52, 6.83) | 0.338 |
| Model 4 | 85 | 77 | 1.50 | (0.42, 5.38) | 0.534 |

^1^ Ordinal logistic mixed model for KOOS symptoms adjusted for repeated measurements (two-level model: measurement/patient)

^2^ Convergence not achieved in three-level mixed model(measurement/patient/surgeon) – surgeon level included as a covariate in two-level mixed model

^3^ Variables which were imbalanced at baseline: Level of education; working status; pre-operative HADS anxiety

Analysis accounting for missing data

|  | Adjustment | N Intervention | N Usual Care | Odds Ratio | 95% CI | P-value |
| --- | --- | --- | --- | --- | --- | --- |
| Analysis using MICE to account for missing data | Model 1 | 89 | 91 | 1.46 | (0.80, 2.66) | 0.222 |
|  | Model 2 | 89 | 91 | 1.45 | (0.79, 2.65) | 0.225 |
|  | Model 3 | 89 | 91 | 1.62 | (0.87, 3.02) | 0.130 |
|  | Model 4 | 89 | 91 | 1.41 | (0.77, 2.59) | 0.261 |
| Analysis using the 10% worse approach^2^ | Model 1 | 89 | 91 | 1.57 | (0.61, 4.00) | 0.349 |
|  | Model 2 | 89 | 91 | 1.57 | (0.61, 4.00) | 0.349 |
|  | Model 3 | 89 | 91 | 1.70 | (0.66, 4.37) | 0.268 |
|  | Model 4 | 89 | 91 | 1.49 | (0.59, 3.79) | 0.401 |
| Analysis using the 10% better approach^3^ | Model 1 | 89 | 91 | 0.85 | (0.31, 2.37) | 0.762 |
|  | Model 2 | 89 | 91 | 0.85 | (0.31, 2.37) | 0.762 |
|  | Model 3 | 89 | 91 | 0.94 | (0.34, 2.60) | 0.900 |
|  | Model 4 | 89 | 91 | 0.75 | (0.28, 2.03) | 0.569 |

^1^ Not multi-level models, single-level ordinal logistic models used for MICE analysis only.

^2^ Missing observations are replaced by category 3 (51 ≤ KOOS symptoms ≤ 75)

^3^ Missing observations are replaced by category 4 (KOOS symptoms >75)

Per protocol analysis

|  | Adjustment | N Intervention | N Usual Care | Odds Ratio | 95% CI | P-value |
| --- | --- | --- | --- | --- | --- | --- |
| Analysis using the Per Protocol principle | Model 1 | 69 | 77 | 2.17 | (0.55, 8.62) | 0.271 |
|  | Model 2 | 69 | 77 | 2.15 | (0.54, 8.59) | 0.276 |
|  | Model 3 | 69 | 77 | 2.65 | (0.66, 10.70) | 0.171 |
|  | Model 4 | 69 | 77 | 1.76 | (0.45, 6.88) | 0.418 |

**Change in treatment effect on KOOS Symptoms between 3, 6 and 12 months**

| Treatment effect (TE)^1^ | Odds ratio | 95% CI | p-value |
| --- | --- | --- | --- |
| How 3 month TE differs from 12 month TE | 0.97 | (0.29, 3.22) | 0.961 |
| How 6 month TE differs from 12 month TE | 0.96 | (0.29, 3.25) | 0.953 |
| How 3 month TE differs from 6 month TE | 0.99 | (0.31, 3.14) | 0.991 |

^1^Derived using contrasts, i.e. difference of appropriate parameters combination assessed in Model 1

# Supplementary material 11.9: HADS Anxiety: Sensitivity and per protocol analyses

The HADS is a secondary outcome. It is comprised of two subscales: Depression and Anxiety. Each sub-scale is derived by summing together 7 Likert-scale type items. Items are rated on a 4-point Likert-type scale ranging from 0 to 3, with higher scores representing greater symptom severity. Each subscale has a score ranging from 0 to 21.

Modelling strategy for analysis of HADS subscales:

- Model 1: Ordinal logistic mixed regression adjusted for stratification variables, pre-operative HADS score and accounting for clustering within patient.
- Model 2: Ordinal logistic mixed regression adjusted for stratification variables, pre-operative HADS score and accounting for clustering within patient and surgeon.
- Model 3: Ordinal logistic mixed regression adjusted for stratification variables, pre-operative HADS score and baseline imbalance variables (Level of education; working status; HADS anxiety) and accounting for clustering within patient.
- Model 4: Ordinal logistic mixed regression adjusted for stratification variables, pre-operative HADS score and whether the patient had received additional physiotherapy during the trial and accounting for clustering within patient.

**Adjusted analysis^1^**

|  | N Intervention | N Usual Care | Odds Ratio | 95% CI | P-value |
| --- | --- | --- | --- | --- | --- |
| Model 2^2^ | 84 | 76 | 2.69 | (0.38, 18.79) | 0.319 |
| Model 3^3^ | 84 | 76 | 3.43 | (0.50, 23.76) | 0.212 |
| Model 4 | 84 | 76 | 2.75 | (0.38, 19.76) | 0.314 |

^1^ Ordinal logistic mixed model for HADS anxiety adjusted for repeated measurements (two-level model: measurement/patient)

^2^ Convergence not achieved in three-level mixed model(measurement/patient/surgeon) – surgeon level included as a covariate in two-level mixed model ^3^ Variables which were imbalanced at baseline: Level of education; working status; pre-operative HADS anxiety

Analysis accounting for missing data

|  | Adjustment | N Intervention | N Usual Care | Odds Ratio | 95% CI | P-value |
| --- | --- | --- | --- | --- | --- | --- |
| Analysis using MICE to account for missing data^1^ | Model 1 | 89 | 91 | 1.28 | (0.58, 2.85) | 0.539 |
|  | Model 2 | 89 | 91 | 1.27 | (0.58, 2.81) | 0.551 |
|  | Model 3 | 89 | 91 | 1.49 | (0.63, 3.54) | 0.364 |
|  | Model 4 | 89 | 91 | 1.31 | (0.58, 2.94) | 0.513 |
| Analysis using the 10% better/worse approach^2^ | Model 1 | 89 | 91 | 2.68 | (0.52, 13.87) | 0.241 |
|  | Model 2 | 89 | 91 | 2.62 | (0.51, 13.56) | 0.251 |
|  | Model 3 | 89 | 91 | 3.34 | (0.64, 17.26) | 0.151 |
|  | Model 4 | 89 | 91 | 3.31 | (0.63, 17.28) | 0.156 |

**^1^** Not multi-level models, single-level ordinal logistic models used for MICE analysis only.

^2^ Median and interquartile range limits all fell into category 1 (HADS anxiety < 7) missing was imputed as category 1

**Per protocol analysis**

|  | Adjustment | N Intervention | N Usual Care | Odds Ratio | 95% CI | P-value |
| --- | --- | --- | --- | --- | --- | --- |
| Analysis using the Per Protocol principle | Model 1 | 68 | 76 | 1.43 | (0.16, 12.48) | 0.749 |
|  | Model 2 | 68 | 76 | 1.35 | (0.15, 12.11) | 0.789 |
|  | Model 3 | 68 | 76 | 2.51 | (0.29, 21.90) | 0.405 |
|  | Model 4 | 68 | 76 | 1.69 | (0.19, 15.46) | 0.642 |

**Change in treatment effect on HADS anxiety between 3, 6 and 12 months**

| Treatment effect (TE)^1^ | Odds ratio | 95% CI | p-value |
| --- | --- | --- | --- |
| How 3 month TE differs from 12 month TE | 0.43 | (0.06, 2.94) | 0.391 |
| How 6 month TE differs from 12 month TE | 0.65 | (0.10, 4.40) | 0.657 |
| How 3 month TE differs from 6 month TE | 1.50 | (0.21, 10.49) | 0.684 |

^1^Derived using contrasts, i.e. difference of appropriate parameters combination assessed in Model 1

# Supplementary material 11.10: HADS Depression: Sensitivity and per protocol analyses

**Adjusted analysis^1^**

|  | N Intervention | N Usual Care | Odds Ratio | 95% CI | P-value |
| --- | --- | --- | --- | --- | --- |
| Model 2^2^ | 84 | 77 | 0.63 | (0.12, 3.20) | 0.579 |
| Model 3^3^ | 84 | 77 | 0.70 | (0.14, 3.41) | 0.656 |
| Model 4 | 84 | 77 | 0.69 | (0.13, 3.51) | 0.651 |

^1^ Ordinal logistic mixed model for HADS depression adjusted for repeated measurements (two-level model: measurement/patient)

^2^ Convergence not achieved in three-level mixed model(measurement/patient/surgeon) – surgeon level included as a covariate in two-level mixed model ^3^ Variables which were imbalanced at baseline: Level of education; Working status; pre-operative HADS anxiety

Analysis accounting for missing data

|  | Adjustment | N Intervention | N Usual Care | Odds Ratio | 95% CI | P-value |
| --- | --- | --- | --- | --- | --- | --- |
| Analysis using MICE to account for missing data | Model 1 | 89 | 91 | 0.76 | (0.35, 1.66) | 0.492 |
|  | Model 2 | 89 | 91 | 0.76 | (0.35, 1.67) | 0.497 |
|  | Model 3 | 89 | 91 | 0.79 | (0.34, 1.82) | 0.574 |
|  | Model 4 | 89 | 91 | 0.78 | (0.35, 1.73) | 0.541 |
| Analysis using the 10% better/worse approach^2^ | Model 1 | 89 | 91 | 0.93 | (0.20, 4.32) | 0.922 |
|  | Model 2 | 89 | 91 | 0.93 | (0.20, 4.32) | 0.922 |
|  | Model 3 | 89 | 91 | 0.94 | (0.21, 4.28) | 0.940 |
|  | Model 4 | 89 | 91 | 1.20 | (0.26, 5.53) | 0.813 |

**^1^** Not multi-level models, single-level ordinal logistic models used for MICE analysis only.

^2^ Median and interquartile range limits all fell into category 1 (HADS depression < 7) missing was imputed as category

|  | Adjustment | N Intervention | N Usual Care | Odds Ratio | 95% CI | P-value |
| --- | --- | --- | --- | --- | --- | --- |
| Analysis using the Per Protocol principle | Model 1 | 68 | 77 | 0.70 | (0.12, 3.97) | 0.685 |
|  | Model 2 | 68 | 77 | 0.70 | (0.12, 3.98) | 0.686 |
|  | Model 3 | 68 | 77 | 1.07 | (0.19, 6.14) | 0.937 |
|  | Model 4 | 68 | 77 | 0.88 | (0.15, 5.07) | 0.884 |

**Per protocol analysis**

**Change in treatment effect on HADS depression between 3, 6 and 12 months**

| Treatment effect (TE)^1^ | Odds ratio | 95% CI | p-value |
| --- | --- | --- | --- |
| How 3 month TE differs from 12 month TE | 0.45 | (0.07, 2.89) | 0.400 |
| How 6 month TE differs from 12 month TE | 1.35 | (0.20, 9.22) | 0.759 |
| How 3 month TE differs from 6 month TE | 3.00 | (0.42, 21.32) | 0.272 |

^1^Derived using contrasts, i.e. difference of appropriate parameters combination assessed in Model 1

# Supplementary material 11.11: Satisfaction with surgery outcome: Sensitivity and per protocol analyses

The Patient Satisfaction Scale is a secondary outcome. It is comprised of four items. Each item is scored on a 4-point Likert scale with response categories consisting of very satisfied (100 points), somewhat satisfied (75 points), somewhat dissatisfied (50 points), and very dissatisfied (25 points). The scale score is the unweighted mean of the scores from the individual items, ranging from 25 to 100 per item (with 100 being most satisfied).

Modelling strategy for analysis of Satisfaction with surgery outcome:

- Model 1: Ordinal logistic regression adjusted for stratification variables and accounting for clustering within patient.
- Model 2: Ordinal logistic regression adjusted for stratification variables and accounting for clustering within patient and surgeon.
- Model 3: Ordinal logistic regression adjusted for stratification variables and baseline imbalance variables (Level of education; working status; HADS anxiety) and accounting for clustering within patient.
- Model 4: Ordinal logistic regression adjusted for stratification variables and whether the patient had received additional physiotherapy during the trial and accounting for clustering within patient.

**Adjusted analysis^1^**

|  | N Intervention | N Usual Care | Odds Ratio | 95% CI | P-value |
| --- | --- | --- | --- | --- | --- |
| Model 2 | 79 | 71 | 1.96 | (0.73, 5.30) | 0.184 |
| Model 3 | 79 | 71 | 2.15 | (0.79, 5.85) | 0.136 |
| Model 4 | 79 | 71 | 1.83 | (0.67, 4.97) | 0.237 |

**^1^** Single level ordinal logistic regression

Analysis accounting for missing data

|  | Adjustment | N Intervention | N Usual Care | Odds Ratio | 95% CI | P-value |
| --- | --- | --- | --- | --- | --- | --- |
| Analysis using MICE to account for missing data^1^ | Model 1 | 89 | 91 | 1.45 | (0.72, 2.93) | 0.296 |
|  | Model 2 | 89 | 91 | 1.46 | (0.72, 2.96) | 0.292 |
|  | Model 3 | 89 | 91 | 1.60 | (0.77, 3.35) | 0.206 |
|  | Model 4 | 89 | 91 | 1.42 | (0.70, 2.88) | 0.336 |
| Analysis using the 10% better/worse approach^2,3^ | Model 1 | 89 | 91 | 2.10 | (0.50, 8.92) | 0.313 |
|  | Model 2 | 89 | 91 | 2.10 | (0.50, 8.92) | 0.313 |
|  | Model 3 | 89 | 91 | 2.34 | (0.55, 9.91) | 0.247 |
|  | Model 4 | 89 | 91 | 1.88 | (0.46, 7.75) | 0.383 |

**^1^** Not multi-level models, single-level ordinal logistic models used for MICE analysis.

^2^ Median and interquartile range limits all fell into category 3 (Satisfaction ≥ 76) missing was imputed as category 3

^3^ Analysed using multi-level ordered logistic regression models.

|  | Adjustment | N Intervention | N Usual Care | Odds Ratio | 95% CI | P-value |
| --- | --- | --- | --- | --- | --- | --- |
| Analysis using the Per Protocol principle | Model 1 | 66 | 71 | 2.10 | (0.72, 6.18) | 0.177 |
|  | Model 2 | 66 | 71 | 2.09 | (0.71, 6.15) | 0.182 |
|  | Model 3 | 66 | 71 | 2.30 | (0.77, 6.90) | 0.137 |
|  | Model 4 | 66 | 71 | 1.92 | (0.64, 5.73) | 0.242 |

**Per protocol analysis^1^**

**^1^**Ordinal logistic regression

**Change in treatment effect on satisfaction with surgery between 3, 6 and 12 months**

| Treatment effect (TE) ^1^ | Odds ratio | 95% CI | p-value |
| --- | --- | --- | --- |
| How 3 month TE differs from 12 month TE | 0.41 | (0.10, 1.67) | 0.214 |
| How 6 month TE differs from 12 month TE | 0.84 | (0.20, 3.62) | 0.816 |
| How 3 month TE differs from 6 month TE | 2.05 | (0.47, 8.92) | 0.339 |

^1^Derived using contrasts, i.e. difference of appropriate parameters combination assessed in Model 1

# Supplementary material 11.12: single item Satisfaction with physiotherapy: Sensitivity and per protocol analyses

The unvalidated single-item satisfaction outcome is also considered to assess the satisfaction with the physiotherapy received by both groups of participants. Based on a 5-item Likert scale ranging from very satisfied (1) to very dissatisfied (5), this variable describes satisfaction with physiotherapy treatment. This is a categorical variable and will be treated as an ordinal variable.

Modelling strategy for analysis of Satisfaction with physiotherapy:

- Model 1: Ordinal logistic regression adjusted for stratification variables and accounting for clustering within patient.
- Model 2: Ordinal logistic regression adjusted for stratification variables and accounting for clustering within patient and surgeon.
- Model 3: Ordinal logistic regression adjusted for stratification variables and baseline imbalance variables (Level of education; working status; HADS anxiety) and accounting for clustering within patient.
- Model 4: Ordinal logistic regression adjusted for stratification variables and whether the patient had received additional physiotherapy during the trial and accounting for clustering within patient.

**Adjusted analysis^1^**

|  | N Intervention | N Usual Care | Odds Ratio | 95% CI | P-value |
| --- | --- | --- | --- | --- | --- |
| Model 2 | 84 | 77 | 0.1 | (0.03, 0.37) | <0.001 |
| Model 3 | 84 | 77 | 0.1 | (0.03, 0.40) | 0.001 |
| Model 4 | 84 | 77 | 0.1 | (0.03, 0.34) | <0.001 |

**^1^** Ordinal logistic mixed model for satisfaction adjusted for repeated measurements

Analysis accounting for missing data

|  | Adjustment | N Intervention | N Usual Care | Odds Ratio | 95% CI | P-value |
| --- | --- | --- | --- | --- | --- | --- |
| Analysis using MICE to account for missing data^1^ | Model 1 | 89 | 91 | 0.30 | (0.15, 0.57) | <0.001 |
|  | Model 2 | 89 | 91 | 0.29 | (0.15, 0.57) | <0.001 |
|  | Model 3 | 89 | 91 | 0.29 | (0.15, 0.57) | <0.001 |
|  | Model 4 | 89 | 91 | 0.28 | (0.15, 0.55) | <0.001 |
| Analysis using the 10% worse approach^2^ | Model 1 | 89 | 91 | 0.16 | (0.06, 0.43) | <0.001 |
|  | Model 2 | 89 | 91 | 0.16 | (0.06, 0.43) | <0.001 |
|  | Model 3 | 89 | 91 | 0.18 | (0.07, 0.48) | 0.001 |
|  | Model 4 | 89 | 91 | 0.16 | (0.06, 0.41) | <0.001 |
| Analysis using the 10% better approach^3^ | Model 1 | 89 | 91 | 0.22 | (0.08, 0.61) | 0.004 |
|  | Model 2 | 89 | 91 | 0.22 | (0.08, 0.61) | 0.004 |
|  | Model 3 | 89 | 91 | 0.23 | (0.09, 0.65) | 0.005 |
|  | Model 4 | 89 | 91 | 0.22 | (0.08, 0.62) | 0.004 |

^1^ Not multi-level models, single-level ordinal logistic models used for MICE analysis only

^2^ Multi-level ordinal logistic regression. Missing observations are replaced by “somewhat satisfied” category

^3^ Multi-level ordinal logistic regression. Missing observations are replaced by “very satisfied” category

|  | Adjustment | N Intervention | N Usual Care | Odds Ratio | 95% CI | P-value |
| --- | --- | --- | --- | --- | --- | --- |
| Analysis using the Per Protocol principle | Model 1 | 69 | 77 | 0.04 | (0.01, 0.16) | <0.001 |
|  | Model 2 | 69 | 77 | 0.04 | (0.01, 0.16) | <0.001 |
|  | Model 3 | 69 | 77 | 0.04 | (0.01, 0.16) | <0.001 |
|  | Model 4 | 69 | 77 | 0.04 | (0.01, 0.16) | <0.001 |

**Per protocol analysis^1^**

^1^Multi-level ordinal logistic regression

**Change in treatment effect on satisfaction with physiotherapy between 3, 6 and 12 months**

| Treatment effect (TE) ^1^ | OR | 95% CI | p-value |
| --- | --- | --- | --- |
| How 3 month TE differs from 12 month TE | 0.54 | (0.17, 1.78) | 0.312 |
| How 6 month TE differs from 12 month TE | 1.32 | (0.42, 4.20) | 0.633 |
| How 3 month TE differs from 6 month TE | 2.44 | (0.75, 7.89) | 0.136 |

^1^Derived using contrasts, i.e. difference of appropriate parameters combination assessed in Model 1

# Supplementary material 12: Serious adverse event data

|  | N  Usual care | N  Intervention |
| --- | --- | --- |
| **TOTAL** | **13** | **8** |
| Manipulation under anaesthetic | 2 | 0 |
| Joint infection | 1 | 1 |
| Suspected infection | 2 | 0 |
| Deep vein thrombosis/pulmonary embolism | 2 | 0 |
| Cellulitis | 1 | 0 |
| Urinary tract infection | 0 | 1 |
| Wound healing problems | 0 | 1 |
| Uncontrolled post-surgical pain | 1 | 0 |
| Post-operative confusion | 0 | 1 |
| Exacerbation of atrial fibrillation during surgery | 0 | 1 |
| Not related to total knee replacement | 4 | 3 |

# Supplementary material 13: Process evaluation data

**QUANTITATIVE DATA FROM INTERVENTION EVALUATION**

| Question | **Category** | **n** | **%** |
| --- | --- | --- | --- |
| How satisfied were you with the structure and format of the classes? | Very satisfied | 45 | 66 |
|  | Satisfied | 16 | 24 |
|  | Dissatisfied | 5 | 7 |
|  | Very dissatisfied | 2 | 3 |
| Do you think that the length of each exercise class was appropriate? | Too long | 1 | 2 |
|  | Just right | 59 | 87 |
|  | Too short | 8 | 12 |
| Do you think that six exercise classes were an adequate number of sessions? | Too many | 2 | 3 |
|  | Just right | 32 | 47 |
|  | Not enough | 34 | 50 |
| Do you think that six weeks after surgery was an appropriate time to start the class? | Too soon | 4 | 6 |
|  | Just right | 58 | 85 |
|  | Too late | 6 | 9 |
| How satisfied were you with the range of exercises included within the exercise class? | Very satisfied | 45 | 66 |
|  | Satisfied | 20 | 29 |
|  | Dissatisfied | 3 | 4 |
|  | Very dissatisfied | 0 | 0 |
|  | **Scale** | **Median** | **IQR** |
| How useful did you find the task-related exercises e.g. walking, strengthening, kneeling etc? | 0-10 (Worst to best) | 9 | 8, 10 |
| How useful did you find the individualised exercises? | 0-10 (Worst to best) | 9 | 8, 10 |
| How helpful do you think the exercise classes were in your recovery after knee replacement? | 0-10 (Worst to best) | 9 | 8, 10 |
| Overall how useful were the exercise classes? | 0-10 (Worst to best) | 9 | 8.5, 10 |
| How useful did you find the written information about the home exercise programme? | 0-10 (Worst to best) | 8 | 8, 9 |
|  | **Yes/no** | **n** | **%** |
| Do you think that the location of the classes was appropriate? | No | 8 | 12 |
|  | Yes | 60 | 88 |
| Have you performed any of the exercises in your home exercise programme since you finished the exercises classes?^1^ | No | 2 | 3 |
|  | Yes | 63 | 93 |
| Have you experienced any complications or problems since you attended the ARENA physiotherapy classes? | No | 59 | 87 |
|  | Yes | 9 | 13 |

^1^3 missing observations (4%)

**FREE-TEXT DATA FROM INTERVENTION EVALUATION**

What aspects of the exercise classes do you think were helpful?

| **Category** | **Examples/reasons** |
| --- | --- |
| Specific exercises (55 comments) | Cycling, treadmill, stairs, digging, kneeling, bed exercises, walking exercises, timed exercises, squatting, broad range of exercises |
| Group format (27 comments) | Peer support provided by groups through the opportunity to meet other and talk with people in a similar stage of recovery, motivation from exercising with others, confidence from exercising with others and seeing how other people are doing |
| Support from physiotherapists (23 comments) | Advice on whether exercises are being performed correctly, how much to exercise, when to stop and what is safe, 1:1 time with a physiotherapist, having the exercises explained and demonstrated , helpful and friendly staff |
| Other (16 comments) | Increased confidence e.g. in daily activities and leisure activities, subsequently joined a gym, provided a reason to leave the house, able to exercise at own pace in classes, helped return to work, taking partner to classes |

**What aspects of the exercise classes do you think were not helpful?**

32 participants said nothing was unhelpful about the classes

| **Category** | **Examples/reasons** |
| --- | --- |
| Class content or structure (17 comments) | Not enough time to talk to other people, not enough classes, not enough support or time with physiotherapist, circuit format confusing, class environment overwhelming, not long enough at each exercise station, not enough time at or between stations, exercises too general, difficult to see other people who are more advanced in their recovery, location of classes not convenient |
| Specific exercises too easy (14 comments) | Bed, walking and stairs exercises too easy because people were already doing these exercises at home. |
| Specific exercises too difficult (8 comments) | Kneeling, squats, stairs, cycling too difficult, daunting or painful |
| Individualised exercises (2 comments) | Not as useful as exercises using equipment, haven’t helped with return to golf or gardening |

Evaluation of home exercise programme

| Would you have liked to receive the information in any other format e.g. video, link to website etc? | 57 thought the paper booklet was appropriate.  8 patients would have preferred a video, website, ipad app or DVD. |
| --- | --- |
| Have you performed any of the exercises in your home exercise programme since you finished the exercises classes? | 63 people had performed the home exercises, 2 people had not. |
| Do you think there were any benefits from performing the exercises? | Improved general function and mobility (n=11)  Helped return to specific activities, such as walking dog, work, gardening, cycling, walking, climbing stairs (n=9)  Psychological benefits – provided motivation and structure, increased confidence in daily activities, made people feel more positive (n=9)  Improved strength in knee (n=9)  Helped with recovery (n=7)  Reduced stiffness (n=6)  Other – people felt they had more energy, less bodily pain, more stamina, prepared people to join a gym (n=5) |
| Are you still performing the exercises in your home exercise programme? | 9 patients not performing their home exercises. Reasons include feeling completed recovered, doing other exercise instead (e.g. walking or cycling), having 1:1 physiotherapy or other co-morbidities. |
| Was there anything that made it more difficult for you to perform the exercises at home? | Lack of equipment at home and/or no access to a gym (n=9)  Difficult to fit into daily routine due to work or other commitments (n=7)  Pain (n=3)  Lack of motivation (n=2)  Post-operative complication (n=2)  Other (n=3)- lack of confidence, slow recovery, problems with other knee |

**FREE-TEXT DATA FROM TRIAL PARTICIPATION EVALUATION**

**Why did you** decide to take part in the study?

| **Category** | **Examples/reasons** |
| --- | --- |
| Altruism (96 comments) | To help patients in the future, to give something back, to improve care, because research is important |
| Personal benefit (48 comments) | Hoping to get physiotherapy classes, wanted to do as much exercise as possible, improve chances at best outcome |
| Other (15 comments) | Study sounded interesting and/or relevant, had spare time, not too much to commit to |

**How did you find taking part in th**e trial? Is there anything that could have improved your experience of taking part?

| **Category** | **Examples/reasons** |
| --- | --- |
| Positive experience (132 comments) | Very good, straight forward to participate, enjoyable, interesting, well organised, questionnaires easy to understand and comprehensive, allocation seemed fair, useful to be able to complete questionnaires over the telephone, teabag and coffee sachet with questionnaires was good |
| Suggested improvements for questionnaire (38 comments) | Fewer questionnaires, shorter questionnaires, less duplication in questions, free-text boxes to allow participants to explain their answers, fewer irrelevant questions |
| Issues with allocation to usual care (4 comments) | Wanted to be allocated to intervention group, felt they missed out on the opportunity for physiotherapy |

**Do you think there were any benefits to taking part in the study?**

33 patients thought there were no benefits to participating in the study

| **Category** | **Examples/reasons** |
| --- | --- |
| Physiotherapy classes (56 comments) | Helped with recovery, better mobility, increased confidence, meeting other people, |
| Altruistic benefits (24 comments) | To help other people in the future, improve outcomes for people with knee replacement |
| Other benefits (29 comments) | Increased awareness of recovery through filling in questionnaires, improved confidence through reflecting on positive improvements when filling in questionnaires, monitoring progress through questionnaires, contact and support from research team, |

**Do you think there were any negatives to taking part in the study?**

105 participants thought there were no negatives to participation

| **Category** | **Examples/reasons** |
| --- | --- |
| Questionnaires (23 comments) | Repetitive questions, some questions not relevant, lengthy, difficult to complete |
| Physiotherapy intervention (9 comments) | Not enough classes, difficulty in travelling to classes |
| Usual care allocation (5 comments) | Wanted to be allocated to physiotherapy group |
